# A Crucial Role of Activin A-Mediated Growth Hormone Suppression in Mouse and Human Heart Failure

**DOI:** 10.1371/journal.pone.0027901

**Published:** 2011-12-28

**Authors:** Noritoshi Fukushima, Katsuhisa Matsuura, Hiroshi Akazawa, Atsushi Honda, Toshio Nagai, Toshinao Takahashi, Akiko Seki, Kagari M. Murasaki, Tatsuya Shimizu, Teruo Okano, Nobuhisa Hagiwara, Issei Komuro

**Affiliations:** 1 Department of Cardiology, Tokyo Women's Medical University, Tokyo, Japan; 2 Global Centers of Excellence (GCOE) Program, Tokyo Women's Medical University, Tokyo, Japan; 3 Institute of Advanced Biomedical Engineering and Science, Tokyo Women's Medical University, Tokyo, Japan; 4 Department of Cardiovascular Medicine, Osaka University Graduate School of Medicine, Osaka, Japan; 5 Department of Cardiovascular Science and Medicine, Chiba University Graduate School of Medicine, Chiba, Japan; Brigham and Women's Hospital, United States of America

## Abstract

Infusion of bone marrow-derived mononuclear cells (BMMNC) has been reported to ameliorate cardiac dysfunction after acute myocardial infarction. In this study, we investigated whether infusion of BMMNC is also effective for non-ischemic heart failure model mice and the underlying mechanisms. Intravenous infusion of BMMNC showed transient cardioprotective effects on animal models with dilated cardiomyopathy (DCM) without their engraftment in heart, suggesting that BMMNC infusion improves cardiac function *via* humoral factors rather than their differentiation into cardiomyocytes. Using conditioned media from sorted BMMNC, we found that the cardioprotective effects were mediated by growth hormone (GH) secreted from myeloid (Gr-1(+)) cells and the effects was partially mediated by signal transducer and activator of transcription 3 in cardiomyocytes. On the other hand, the GH expression in Gr-1(+) cells was significantly downregulated in DCM mice compared with that in healthy control, suggesting that the environmental cue in heart failure might suppress the Gr-1(+) cells function. Activin A was upregulated in the serum of DCM models and induced downregulation of GH levels in Gr-1(+) cells and serum. Furthermore, humoral factors upregulated in heart failure including angiotensin II upregulated activin A in peripheral blood mononuclear cells (PBMNC) via activation of NFκB. Similarly, serum activin A levels were also significantly higher in DCM patients with heart failure than in healthy subjects and the GH levels in conditioned medium from PBMNC of DCM patients were lower than that in healthy subjects. Inhibition of activin A increased serum GH levels and improved cardiac function of DCM model mice. These results suggest that activin A causes heart failure by suppressing GH activity and that inhibition of activin A might become a novel strategy for the treatment of heart failure.

## Introduction

Heart failure is a major cause of mortality in many countries. Infusion of bone marrow-derived mononuclear cells (BMMNC) is expected as a novel treatment of heart failure. Animal experiments and clinical trials have shown that BMMNC infusion ameliorates cardiac dysfunction after acute myocardial infarction and chronic myocardial ischemia [1]–[4]. Although the outcomes vary among trials, recent meta-analyses revealed that cardiac function slightly improves following BMMNC infusion for ischemic heart diseases [5], [6]. Bone marrow cells were reported to be incorporated into the damaged myocardium and to differentiate into various cell types including cardiomyocytes [7]. However, whether bone marrow-derived stem cells can differentiate into many cardiomyocytes is still an open question [8]. There are many reports indicating that transplantation of various types of stem cells improves the cardiac function of ischemic hearts, mainly by paracrine factors which induce angiogenesis and cardioprotection [9]–[11]. Since the effects of BMMNC infusion for non-ischemic cardiomyopathy remain unknown, we examined whether BMMNC infusion also improves cardiac function of non-ischemic cardiomyopathy.

## Results

### Preparation of non-ischemic dilated cardiomyopathy (DCM) mice

**Figure 1:**
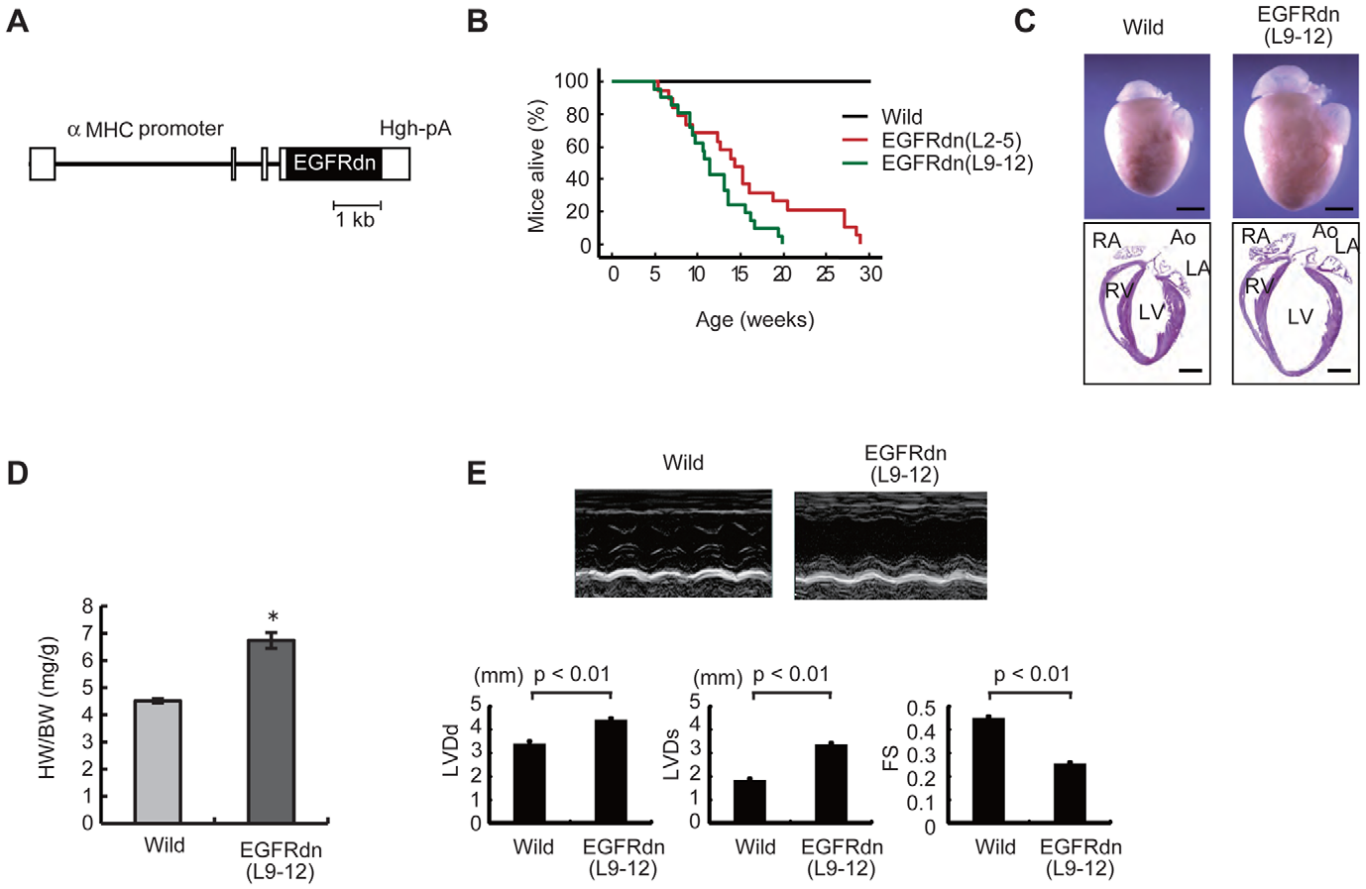
Transgenic overexpression of EGFRdn in the heart causes progressive heart failure. (A) Schematic representation of the cDNA construct used to generate EGFRdn mice. The construct contains an αMHC promoter, human EGFRdn cDNA and a human *growth hormone* polyadenylation signal (Hgh-pA). (B) Kaplan-Meier survival curves for wild-type (*n* = 62) and EGFRdn (L2–5, *n* = 19; L9–12, *n* = 21) mice, showing a significant reduction in the survival rates in EGFRdn mice (log rank test, *P*<0.0001). (C) Gross morphology of whole hearts (upper panels) and longitudinal sections (lower panels) of hearts from wild-type and EGFRdn mice (L9–12) at 6 weeks of age. Ao, aorta; LA, left atrium; LV, left ventricle; RA, right atrium; RV, right ventricle. Scale bars: 2 mm. (D) Heart-to-body weight ratios (HW/BW) of wild-type (*n* = 9) and EGFRdn (L9–12, *n* = 7) mice at 6 weeks of age. *P<0.01. (E) Echocardiographic analysis. The upper photographs show representative M-mode images. The lower graphs show the left ventricular diastolic and systolic dimensions and FS of 8 week-old EGFRdn mice (L9–12) (*n* = 23) and age-matched wild-type mice (*n* = 10). LVDd, left ventricular diastolic dimension; LVDs, left ventricular systolic dimension. Data are means ± s.e.m.

Two kinds of non-ischemic DCM mice were used. The first model was generated by transgenic overexpression of a mutant epidermal growth factor receptor (EGFR) with C-terminal truncation (EGFRdn). The expression of mutant EGFRdn is activated by the cardiomyocyte-specific α-myosin heavy chain (αMHC) promoter (Figure 1A, Figure S1). EGFRdn mice exhibited heart failure and died at 5–30 weeks of age (Figure 1B). Gross inspection of the EGFRdn hearts showed global chamber dilatation with marked wall thinning (Figure 1C). The heart/body weight ratio was approximately 1.5-fold higher at 6 weeks of age in EGFRdn mice than in wild-type mice (Figure 1D). Echocardiography showed a significant decrease in the fractional shortening (FS) together with chamber dilatation (Figure 1E). In the second model, cardiomyopathy was induced by intraperitoneal injection of doxorubicin in wild-type mice. Doxorubicin-induced cardiomyopathy (DOX) mice showed marked dilatations of the left ventricular diastolic and systolic dimensions, and reduction of cardiac function (Figure S2).

### Intravenous infusion of BMMNC transiently improved the cardiac function in DCM mice

**Figure 2:**
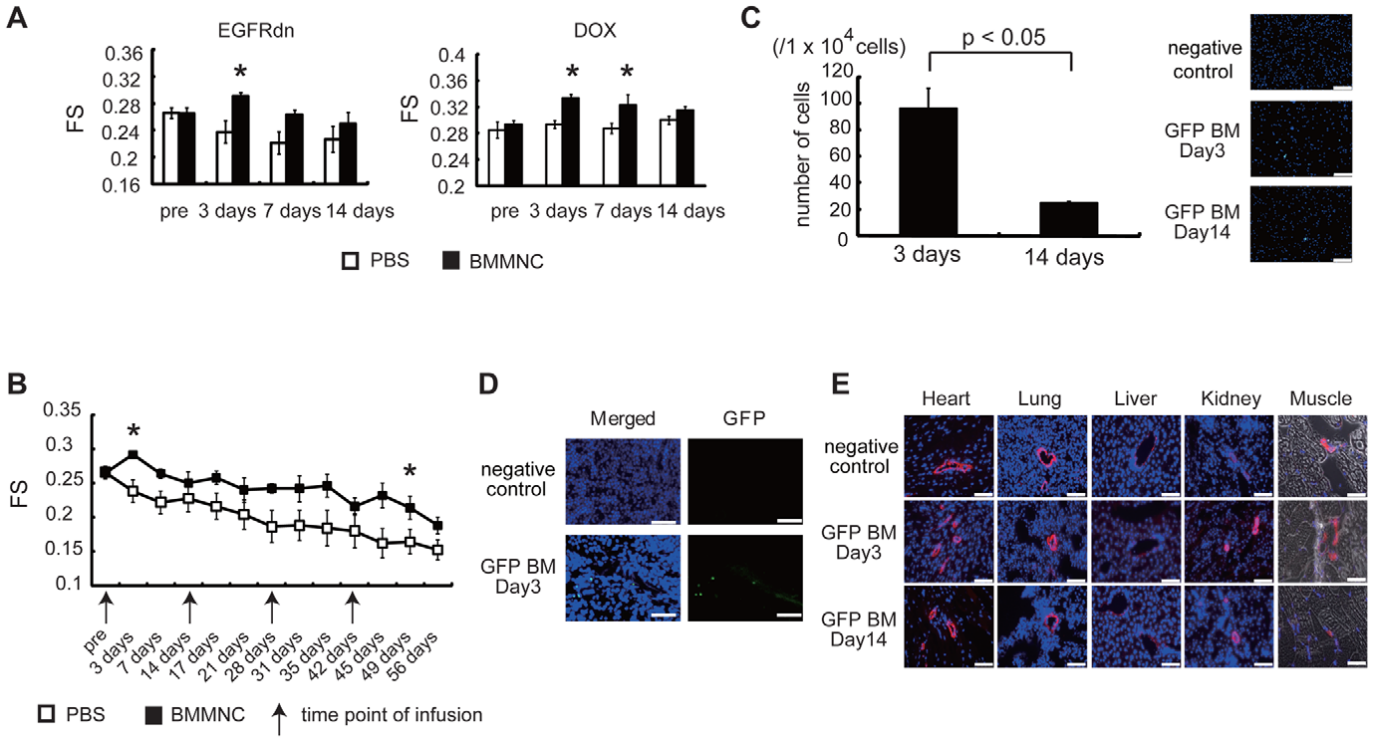
BMMNC infusion transiently improved the cardiac function of DCM mice. (A) Echocardiographic analysis. Transient improvements of FS were observed at 3 d in the BMMNC-treated group, but not the control (PBS) group, in EGFRdn mice (left), and at 3 and 7 d in DOX-treated mice (right). *p<0.05 versus PBS (*n* = 8 per group). (B) Repeated-infusion experiments. BMMNC were infused every 2 weeks. A similar pattern of improvement in FS was observed after each infusion. *p<0.05 versus PBS (*n* = 8 per group). (C–E) Immunohistochemical analysis. (C) Left, the number of GFP-positive BMMNC in peripheral blood (*n* = 3). Right, photomicrographs of peripheral blood. Nuclei were stained with Hoechst 33258 (blue). Scale bars, 75 µm. (D) Images of the spleen 3 d after infusion. Many GFP-positive cells were observed in the spleen (lower photographs). Upper photographs, negative control. Nuclei were stained with Hoechst (blue color). Scale bars, 25 µm. (E) No GFP-positive cells were observed in any organs. Upper photographs, negative control. Middle and lower photographs, images taken at 3 and 14 d, respectively, after infusion. The vessels were stained with smooth muscle cell actin (red). Nuclei were stained with Hoechst 33258 (blue). The photographs of muscle are merged fluorescent and phase-contrast images. Scale bars, 75 µm. Data are means ± s.e.m.

BMMNC (2.0×10^7^ cells) were isolated from wild-type healthy mice and intravenously infused *via* the tail veins to 8-week-old EGFRdn mice and 11-week-old DOX mice. An equal volume of PBS was infused into control mice. Three days after infusion, echocardiography showed that the FS was significantly improved in BMMNC-treated EGFRdn (Figure 2A) and DOX (Figure 2A) mice, compared with the respective controls. However, these effects were lost by 14 d after infusion (Figure 2A). When the infusion was repeated every 2 weeks, cardiac function showed improvements for >50 d (Figure 2B).

Although infusion of BMMNC is not promising for the treatment of heart failure, we may be able to apply alternative treatment if we understand the underlying mechanisms of beneficial effects of BMNNC infusion. To elucidate the mechanisms, we infused BMMNC derived from GFP mice. Although many GFP-positive cells were observed in the peripheral blood and the spleen at 3 d after infusion (Figure 2C, D), none were found in the heart, lung, liver, kidney or skeletal muscle (Figure 2E). At day 14, few GFP-positive cells were observed even in the peripheral blood (Figure 2C). This was consistent with the observation that BMMNC infusion improved cardiac function at day 3, but not at day 14. These results suggest that BMMNC infusion improves the systolic function of DCM mice not by transdifferentiation of BMMNC into cardiomyocytes but probably by humoral factors secreted from BMMNC. Size of each cardiomyocyte was larger in BMMNC-infused EGFRdn mice than in PBS-infused EGFRdn mice when infusions were repeated every 2 weeks for 8 weeks (i.e., 4 injections) (Figure S3). There were no changes in capillary density or the number of apoptotic cells in the heart between the BMMNC-infused group and the control group (data not shown).

### BMMNC-derived conditioned medium (CM) improved cardiomyocyte contractility

**Figure 3:**
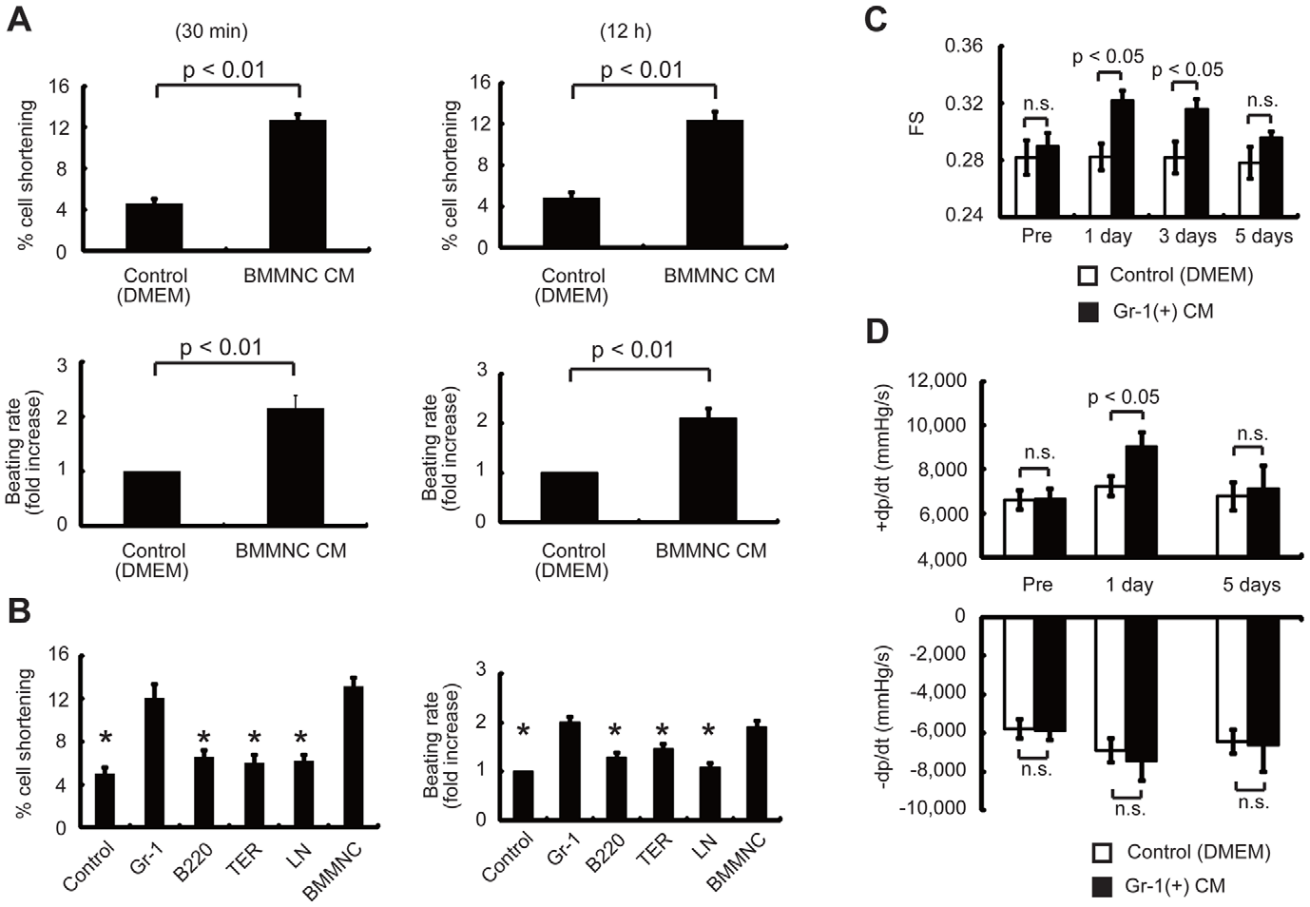
BMMNC-derived CM directly affects cardiomyocyte contractility. (A) Cell shortening and the beating rate of neonatal rat cardiomyocytes were significantly increased after exposure to CM from BMMNC compared with the control (*n* = 26 cells per group). The left and right graphs show the results at 30 min and at 12 h after treatment, respectively. Upper graph, cell shortening. Lower graph, beating rate. (B) CM from Gr-1(+) cells improved the cell shortening and increased the beating rate similar to that achieved by CM from BMMNC (*n* = 27 per group). (C, D) Effects of CM from Gr-1 cells on cardiac function *in vivo*. (C) Echocardiographic analysis (*n* = 7). The infusion of CM from Gr-1(+) cells significantly improved the FS of DOX mice at 1 and 3 d. (D) Infusion of CM from Gr-1(+) cells significantly improved the +dp/dt of DOX mice at 1 d, *in vivo* (*n* = 7). n.s., not significant. Data are means ± s.e.m.

To elucidate whether factors secreted from BMMNC were involved in their beneficial effects on cardiac function, we first examined the effects of CM from BMMNC on the contractility of cultured cardiomyocytes of neonatal rats. After serum starvation for 12 h, cardiomyocytes were challenged with culture medium conditioned by BMMNC. Cell shortening was significantly enhanced and beating rate was markedly increased at 30 min and at 12 h after starting culture with the CM, compared with those in untreated cells (Figure 3A), suggesting that BMMNC secrete factors that positively affect cardiomyocyte contractility. Flow cytometric analysis revealed that BMMNC consisted of several cell populations including myeloid (Gr-1(+) cells, ∼40%), erythroid (TER119(+) cells, ∼20%), and lymphoid cells (B220(+) cells, ∼20%) (Figure S4). The individual cell populations, including the lineage-negative population of cells, were sorted by magnetic beads. The isolated cells were 0.8×10^7^ Gr-1(+) cells, 0.4×10^7^ B220(+) cells, 0.2×10^7^ TER(+) cells, and 0.1×10^7^ lineage-negative cells from 2.0×10^7^ BMMNC. When CM was collected from each population and added to cardiomyocytes starved for 12 h, only the CM from Gr-1(+) cells significantly enhanced cell shortening and increased the beating rate (Figure 3B), suggesting that Gr-1(+) cells mainly contribute to BMMNC-mediated improvements in cardiomyocyte contractility. CM from Gr-1(+) cells or BMMNC isolated from wild-type mice also induced significant hypertrophy of cardiomyocytes (Figure S5). We next examined the effects of CM from Gr-1(+) cells on DOX mice. At 1 and 3 d after the infusion of CM from Gr-1(+) cells, FS was significantly improved, as with infusion of BMMNC (Figure 3C). Furthermore, +dp/dt, as determined by catheterization of the left ventricle, was also improved at 1 d after the infusion, as compared with the control group (Figure 3D). Collectively, these results indicate that factors secreted from Gr-1(+) cells are responsible for BMMNC-induced improvements in cardiac function in DCM mice.

### Analysis of factors secreted from Gr-1(+) cells

**Figure 4:**
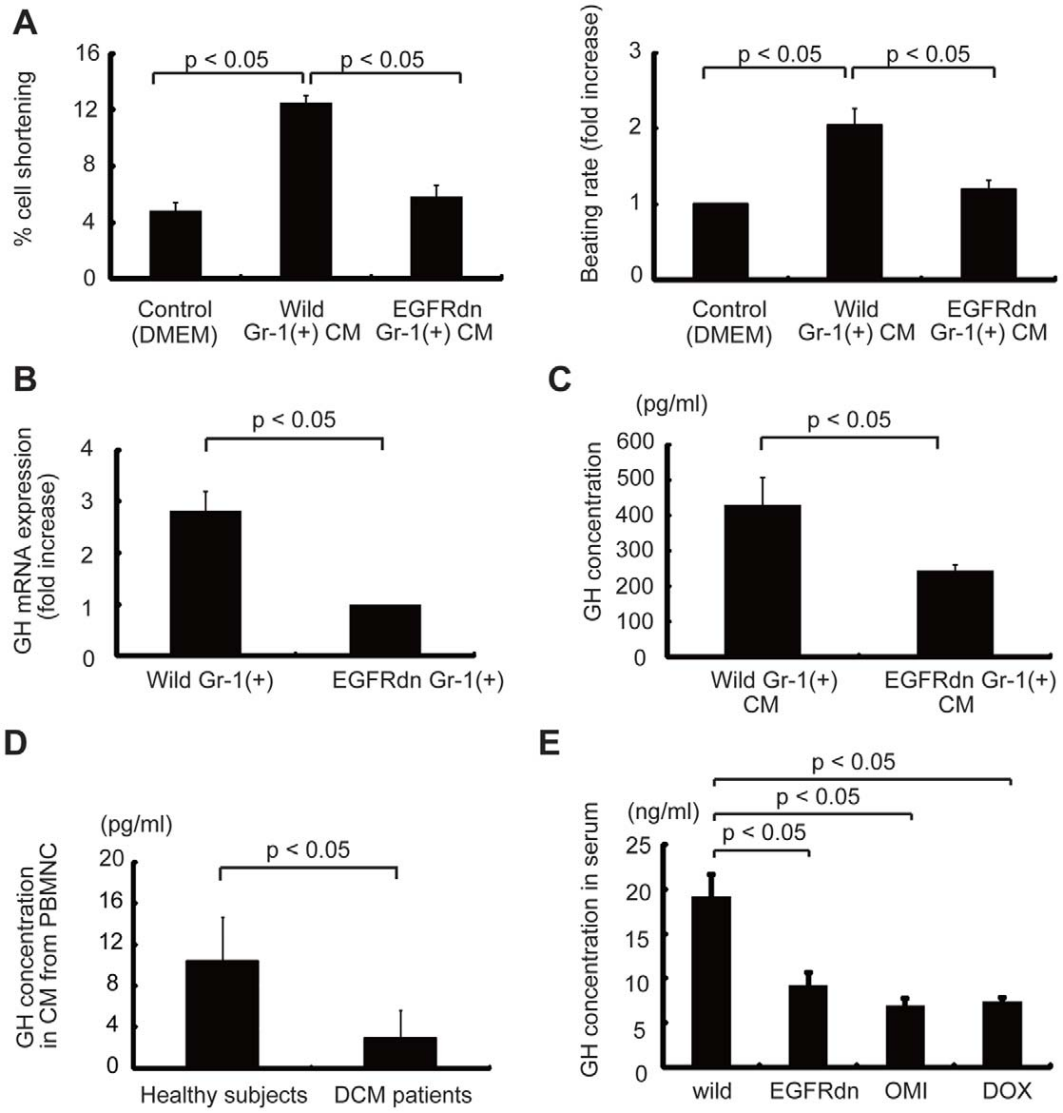
Analysis of secreted factors. (A) CM from Gr-1(+) cells from wild-type mice significantly improved the cell shortening and increased the beating rate in neonatal rat cardiomyocytes, as compared with CM from Gr-1(+) cells from EGFRdn mice. Left graph, cell shortening (*n* = 24 cells per group). Right graph, beating rate (*n* = 24 cells per group). (B) Quantitative RT-PCR analysis of GH mRNA in Gr-1(+) cells isolated from wild-type mice and EGFRdn mice (*n* = 4). (C, D) GH concentrations in (C) CM from Gr-1(+) cells isolated from wild-type mice and EGFRdn mice (*n* = 4) and (D) CM from PBMNC isolated from healthy (*n* = 11) and DCM subjects (*n* = 10). (E) GH concentration in serum from several mouse models of heart failure (*n* = 4). Data are means ± s.e.m.

The CM from wild-type Gr-1(+) cells significantly enhanced cell shortening and increased the beating rate, while CM from EGFRdn Gr-1(+) cells had marginal effects (Figure 4A). This suggests that the factors that improve cardiomyocyte contractility are more abundant in cells of wild-type mice than cells of EGFRdn mice. We next performed DNA microarray analysis to identify the factors involved in these effects. Twenty three genes showed enhanced expression in Gr-1(+) cells from wild-type mice compared with EGFRdn mice (Table 1). The gene which showed the largest difference between two types of mice was growth hormone (GH). The reduced expression of GH in Gr-1(+) cells from EGFRdn mice was confirmed by quantitative RT-PCR and ELISA (Figure 4B, C). GH levels were also lower in CM from Gr-1(+) cells isolated from old myocardial infarction (OMI) mice and DOX mice (Figure S6) than in CM from wild-type mice. Consistent with the downregulation of GH secretion from Gr-1(+) cells of heart failure mice, the serum GH levels were also lower in models of heart failure such as DOX, EGFRdn and OMI mice than in wild-type mice (Figure 4E).

**Table 1:** DNA microarray analysis.

The fold increase	Gene symbol
4.9	Gh
4.3	Pdgfd
3.9	Figf
3.4	Tslp
3.2	Socs2
3.1	Lta
3.0	Bmp1
2.9	Il33
2.8	Ccl27a
2.7	Fgf20
2.6	Angpt1
2.5	Cxcl9
2.4	Il13
2.3	Fam3b
2.3	Il31
2.3	Gm6590
2.2	Spred1
2.2	Cmtm8
2.1	Kitl
2.1	Mif
2.1	Grem2
2.1	Il17d
2.1	Gdf10
2.0	Cxcl5

Each number indicates the fold-increase of gene expression in Gr-1(+) cells isolated from wild-type mice compared with those from EGFRdn mice.

### Critical role of GH in Gr-1(+) cell-mediated cardioprotection

**Figure 5:**
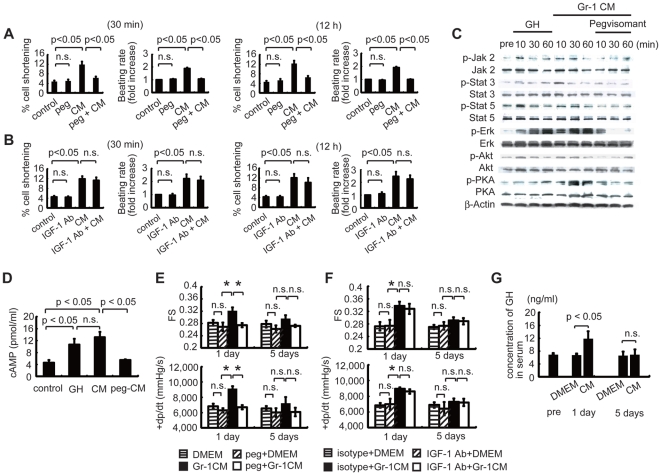
GH mediates the cardioprotective effects of Gr-1(+) cell-derived CM. (A) Pegvisomant (PEG) treatment inhibited the Gr-1(+) cell CM-mediated improvements cardiomyocyte cell shortening and beating rate at 30 min and at 12 h after treatment (*n* = 27 cells per group). Left graphs, cell shortening; right graphs, beating rate. (B) Anti-IGF-1 antibody failed to affect the Gr-1(+) cell CM-mediated improvements in cell shortening or beating rate at 30 min or at 12 h after treatment (*n* = 23 cells per group). Left graphs, cell shortening; right graphs, beating rate. (C) GH and CM from Gr-1(+) cells phosphorylated Akt, Erk, Jak2, Stat3/5 and PKA in cardiomyocytes (*n* = 3), which was inhibited by pegvisomant (*n* = 3). (D) GH (500 pg/ml) and CM from Gr-1(+) cells increased the cAMP concentration in cardiomyocytes (*n* = 5), which was inhibited by pegvisomant (*n* = 5). (E, F) Cardiac function analysis by echocardiography (upper graphs, *n* = 8) and catheterization (lower graphs, *n* = 8). Pegvisomant (E), but not anti-IGF-1 antibody (F), inhibited the improvements in FS and +dp/dt elicited by the infusion of CM from Gr-1(+) cells. *p<0.05 (*n* = 8). (G) Serum GH concentrations in DOX mice treated with CM from Gr-1(+) cells (*n* = 4 per group). The infusion of CM from Gr-1(+) cells from wild-type mice increased the serum GH concentration at 1 d, but not at 5 d. Data are means ± s.e.m.

We examined the role of GH in the effects of Gr-1(+) cell-derived CM using pegvisomant, a specific inhibitor of the GH receptor [12]. Treatment with pegvisomant abolished the enhanced cell shortening and the increased beating rate induced by CM from Gr-1(+) cells (Figure 5A), while the anti-IGF-1 antibody had no effects (Figure 5B). These results suggest that Gr-1(+) cells improved the cardiomyocyte contractility *via* GH, but not *via* IGF-1 *in vitro*. CM from Gr-1(+) cells activated various signaling molecules, including Akt, extracellular signal-regulated kinase (Erk) 1/2, Janus kinase (Jak) 2, signal transducers and activators of transcription (Stat) 3/5 and protein kinase A (PKA) in cardiomyocytes (Figure 5C), and these effects were completely abolished by pegvisomant (Figure 5C). The addition of GH (500 pg/ml), a concentration equivalent to that in the CM from wild-type Gr-1(+) cells, activated the same signaling molecules (Figure 5C), suggesting that CM from Gr-1(+) cells activates Akt, Erk1/2, Jak2, Stat3/5 and PKA through the GH receptor signaling. Furthermore, the CM from Gr-1(+) cells, as well as GH, increased the amount of cyclic AMP (cAMP) in cardiomyocytes, which was also inhibited by pegvisomant (Figure 5D). The improvements in cardiac function induced by CM from Gr-1(+) cells were also abolished by treatment with the GH inhibitor (Figure 5E), whereas the anti-IGF-1 antibody had no effects (Figure 5F). Furthermore, the infusion of CM from Gr-1(+) cells increased the GH levels in serum of DCM mice (Figure 5G). These results suggest that Gr-1(+) cells improve the cardiac contractility *in vivo* also through GH. The BMMNC-mediated improvement in cardiac function of OMI mice was also affected by treatment with pegvisomant (Figure S7), suggesting that GH in BMMNC might have the therapeutic effects on heart failure caused by various etiologies.

Since Stat 3 is one of the important downstream targets of the GH receptor in cardiomyocytes (Figure 5C), we examined the direct effects of GH in CM from Gr-1(+) cells on cardiomyocytes *in vivo* in transgenic mice overexpressing a dominant-negative mutant of STAT3 (STAT3dn) under the control of an αMHC promoter [13]. The Gr-1(+) cell CM-mediated improvements in cardiac function were not observed in DOX-treated STAT3dn mice (Figure S8), indicating that the CM improves cardiac function through activation of STAT3 in cardiomyocytes.

### Upregulation of activin A in heart failure inhibits GH expression in Gr-1(+) cells

**Figure 6:**
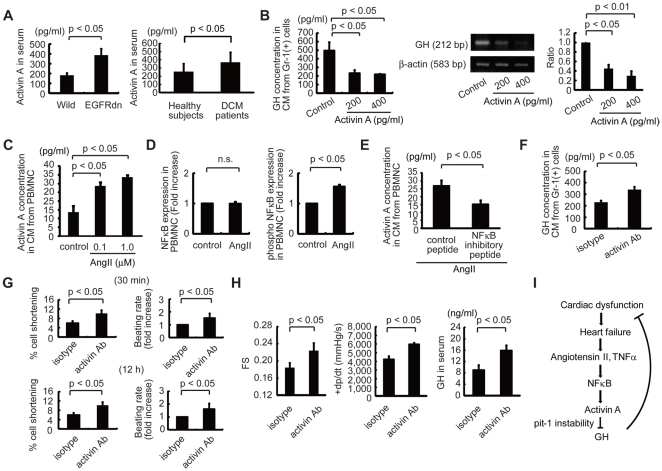
Regulatory mechanisms of GH in heart failure. (A) The serum activin A concentration was higher in EGFRdn mice (left, *n* = 5) and in DCM patients (right, *n* = 10) than in wild-type mice (*n* = 5) and healthy subjects (*n* = 11). (B) Activin A downregulated GH mRNA expression in Gr-1(+) cells and GH protein levels in Gr-1(+) cell CM. Left graph, GH protein concentration; middle photographs, representative semi-quantitative RT-PCR images; right graph, GH mRNA expression (*n* = 3). (C, D) AngII upregulated activin A secretion (C, *n* = 4) and phosphorylated NFκB expression (D, *n* = 5) in wild-type PBMNC. (D) Left graph, total NFκB; right graph, phosphorylated NFκB. (E) Inhibition of NFκB [50 µM; NFκB p65 (Ser276) inhibitory peptide] suppressed AngII (10 µM)-mediated upregulation of activin A in CM derived from wild-type PBMNC (*n* = 5). Isotype peptide was used as control. (F) The GH concentration in CM from EGFRdn Gr-1(+) cells (*n* = 5) was significantly increased by treatment with an anti-activin A antibody (*n* = 5). (G) Effects of anti-activin A antibody treatment on cell shortening and the beating rate of cardiomyocytes induced by CM from Gr-1(+) cells isolated from EGFRdn mice (*n* = 18 cells per group). (H) Treatment with the anti-activin A antibody improved the cardiac function of EGFRdn mice. Left graph, echocardiography (*n* = 7). Middle graph, miller catheter results (*n* = 7). Right graph, serum GH concentration in EGFRdn mice after antibody treatment (*n* = 7). Data are means ± s.e.m. (I) Proposed mechanism underlying impaired GH expression by activin A in heart failure.

The expression of the GH gene has been reported to be regulated by transcription factors including pituitary transcription activator-1 (pit-1) [14], [15], and activin A has been reported to downregulate GH expression by reducing the stability of pit-1 [16]. Since activin A in the peripheral blood of heart failure patients has been reported to be upregulated compared with that in healthy controls [17], we investigated the role of activin A in the downregulation of GH in Gr-1(+) cells. Serum activin A levels were significantly higher in EGFRdn mice than in wild-type mice (Figure 6A), and were also elevated in other murine models of heart failure, including the OMI and DOX models (Figure S9). When Gr-1(+) cells were cultured with 400 pg/ml of activin A, a concentration equivalent to that in the peripheral blood of DCM mice, mRNA and protein levels of GH were significantly downregulated (Figure 6B), suggesting that activin A might be a key mediator of the reduced expression of GH in the Gr-1(+) cells of DCM mice. Furthermore, the serum activin A levels were remarkably higher in DCM patients (Table S1) than in healthy subjects (Figure 6A), while the GH levels in CM from peripheral blood mononuclear cells (PBMNC) of DCM patients was lower than that in healthy subjects (Figure 4D), suggesting that the higher activin A levels might also inhibit GH expression in heart failure patients. A recent study showed that PBMNC are a major source of activin A in heart failure [17]. Since many humoral factors are known to contribute to the pathophysiology of heart failure [18], we examined whether humoral factors upregulated in heart failure might regulate activin A expression. Angiotensin II (AngII) (Figure 6C) and tissue necrosis factor-alpha (TNFα) (Figure S10A) increased the activin A levels in CM of PBMNC in a dose-dependent manner. Consistent with the previous reports [19], AngII and TNFα activated NFκB in the PBMNC (Figure 6D and Figure S10B) and AngII- and TNFα-induced upregulation of activin A in PBMNC were inhibited with a NFκB inhibitory peptide (Figure 6E and Figure S10C).

### Inhibition of activin A in heart failure increases GH levels and improves cardiac function

To elucidate the role of activin A in EGFRdn mice, anti-activin A antibody was injected intraperitoneally for 2 weeks, with an alternate-day treatment regimen. Inhibition of activin A significantly increased GH protein levels in the CM from Gr-1(+) cells (Figure 6F). Furthermore, when neonatal rat cardiomyocytes were cultured with CM from Gr-1(+) cells isolated from anti-activin A antibody-treated EGFRdn mice, cell shortening was enhanced and the beating rate was increased significantly, as compared with CM from Gr-1(+) cells without antibody treatment (Figure 6G). Consistent with the upregulation of GH levels in Gr-1(+) cells by anti-activin A antibody treatment, the serum GH levels in EGFRdn mice were also increased (Figure 6H). Furthermore, FS and +dp/dt in EGFRdn mice treated with anti-activin A antibody were markedly improved compared with EGFRdn mice treated with isotype control (Figure 6H). Collectively, these results strongly suggest that inhibition of activin A improves cardiac function in non-ischemic DCM mice by restoring GH levels.

## Discussion

Functional benefits of BMMNC infusion have been reported in human with ischemic heart diseases [2],[20]. Although we also observed the improvement of cardiac function of DCM model mice by BMMNC infusion, no engraftment of infused BMMNC was observed in the heart. At 3 d after infusion, BMMNC were only observed in the peripheral blood and spleen, but not in the heart, and very few GFP-positive cells were observed at 14 d even in the peripheral blood. This is consistent with the observations that BMMNC infusion only transiently improved cardiac function after infusion. These findings suggest that BMMNC improve cardiac function *via* humoral factors rather than *via* transdifferentiation into cardiomyocytes.

GH plays important roles in the protection of various tissues as well as the growth and development of many organs and whole body [21]. Serum GH levels have been reported to be low in patients with congestive heart failure [22]. Recent animal studies have demonstrated that GH treatment improves cardiac functions [23], [24]. The growth and protection of cardiomyocytes are regulated by various kinases such as Akt, Erk and Jak/Stat, and many studies have demonstrated that activation of Akt and Erk induces cardiac hypertrophy [25], [26] and prevents cardiomyocytes from stress-induced apoptosis [27]. Transgenic mice with cardiac-specific overexpression of the *stat3* gene were reported to show marked ventricular hypertrophy [28], while the cardioprotective effects of several cytokines including granulocyte colony-stimulating factor were reduced in mice with cardiac-specific expression of dominant-negative *stat3* [29]. In this study, we showed that GH produced by Gr-1(+) cells activated Akt, Erk, Jak2, Stat3/5 and PKA, and increased the levels of cAMP in neonatal rat cardiomyocytes (Figure 5C, D). GH has been reported to increase cAMP and activate PKA in reproductive organs by still-unknown mechanisms [30]. Here, we found that the beneficial effects of CM from Gr-1(+) cells on cardiac function were inhibited in cardiac-specific STAT3dn mice, suggesting that GH secreted by Gr-1(+) cells directly affects cardiomyocyte contractility. It has been reported that GH exerts some functions through the induction of IGF-1 expression [31], [32], and IGF-1 also promotes several cardioprotective effects in part by activating the Akt/phosphatidylinositol 3-kinase pathway [33], [34]. In the present study, the specific GH receptor inhibitor, but not anti-IGF-1 antibody, attenuated the improvements of cardiac contractility by the treatment of CM from Gr-1(+) cells in vitro (Figure 5A, B) and in vivo (Figure 5E, F). These findings suggest the effects of Gr-1(+) cells-derived CM on cardiac function of DCM mice mainly depend on GH rather than IGF-1.

It has been reported that the expression of GH gene is regulated by pit-1 at the transcriptional level [14], [15] and that activin A destabilizes pit-1 by phosphorylation [16]. Consistent with a previous report showing higher serum activin A levels in heart failure patients than in healthy controls [17], we found that serum levels of activin A were increased while GH levels in PBMNC CM were decreased in DCM patients. Similarly, the activin A levels were higher in the peripheral blood of DCM mice than in wild-type mice and activin A inhibited the production of GH in Gr-1(+) cells *in vitro*. These findings suggest that activin A, which is upregulated in heart failure, inhibits GH expression in various tissues/cells, including BMMNC. Treatment with anti-activin A antibody restored GH levels in Gr-1(+) cells and serum of EGFRdn mice and improved cardiac function, suggesting that normalizing the GH levels by inhibiting activin A is a novel therapeutic strategy for heart failure. Since many humoral factors such as AngII and TNFα are upregulated in heart failure and increased activin A expression by activating NFκB, the molecules that modulate NFκB activation might be also therapeutic targets to restore GH levels. On the other hand, anti-activin A treatment also increased expression levels of GH mRNA in the pituitary (N.F. K.M., unpublished data), suggesting that upregulation of activin A in heart failure might inhibit the expression of GH not only in Gr-1(+) cells but also in the pituitary, and that anti-activin A treatment might improve cardiac function of DCM mice in part by restoring GH expression in the pituitary.

The effects of GH on heart failure have been examined in many animal experiments and clinical trials [35]. A recent meta-analysis revealed that GH treatment improved several clinical parameters including left ventricular end-diastolic dimension, ejection fraction and New York Heart Association functional class [36]. Conversely, non-response to GH treatment for heart failure has been ascribed to GH resistance [37]. In patients with cardiac cachexia, GH levels were reported to be enhanced when compared with non-cachectic patients and normal subjects [38]. In this study, GH levels in heart failure mice and patients were significantly lower than those in healthy control subjects. Moreover, GH derived from Gr-1(+) cells improved cardiac function of heart failure animals, suggesting that our models were in a non-cachectic state and non-cachectic patients of heart failure might be suitable for GH treatment. Because of only temporary improvements in cardiac function (Figure 2A), bone marrow cell infusion might not be an appropriate treatment for heart failure, however inhibition of activin A and enhancement of GH levels might offer novel therapeutic strategies for heart failure.

We used EGFRdn for DCM model mice in this study. It has been reported that cardiac-specific mutant of *ErbB2*, a member of the EGFR/erbB family, shows a severe dilated cardiomyopathy in mice [39]. In the clinical setting, trastuzumab, an anti-cancer agent, is humanized monoclonal antibody that targets the extracellular domain of the human epidermal growth factor receptor 2 and the use of trastuzumab demonstrated an unexpectedly high incidence of both asymptomatic and symptomatic cardiomyopathy. EGFRdn is a compatible DCM model mouse, resembling the cardiotoxic effects observed in patients treated with trastuzumab [40], [41].

There is a limitation in this study. We examined the surface area of neonatal rat cardiomyocytes after the treatment with CM from Gr-1(+) cells or BMMNC as an index for cardiac hypertrophy. However, the surface area not only depends on cell volume, but also on the degree of adhesion and spreading on the culture dishes.

## Materials and Methods

**Ethics Statement.** The ethical committee of Tokyo Women's Medical University reviewed and approved the study protocol (approval ID: 1795). The study was conducted in accordance with the Declaration of Helsinki. We obtained informed consent from the all patients and the all healthy subjects by written before inclusion in this study.

**Animals.** Wild-type mice (C57BL/6) were purchased from Japan SLC. Adult GFP transgenic mice (C57BL/6) were a kind gift from Dr. M. Okabe (Osaka University). Cardiac-specific dominant-negative STAT3 mice were a kind gift from Dr. K. Yamauchi-Takihara (Osaka University). Neonatal Wistar rats (0–1 d old) were purchased from Saitama Experimental Animals Supply. All protocols were approved by the Institutional Animal Care and Use Committee of Tokyo Women's Medical University and Chiba University. The approval IDs for the animal experiments were 11–34 in Tokyo Women's Medical University and A21–178 in Chiba University. Doxorubicin (10 mg/kg body weight) was intraperitoneally injected into wild-type male mice (C57BL/6) once-weekly at weeks 7 and 8 after birth. After both Doxorubicin injections, the mice were reared for a further 2 weeks, and the surviving mice were used for experiments. Myocardial infarction models were prepared using wild-type male mice (C57BL/6) as previously described [11]. Serum and Gr-1(+) cells were isolated 4 weeks after inducing myocardial infarction (11 weeks of age).

**Generation of EGFRdn mice.** The C-terminal 533 amino acids [42] were deleted from the full-length human *EGFR* cDNA (a gift from Professor T. Kadowaki, The University of Tokyo) by introducing a stop codon (TGA) after the R677 codon by site-directed mutagenesis. The truncated *EGFR* (*EGFRdn*) cDNA was then subcloned into the *αMHC* promoter-containing expression vector (a gift from Professor J. Robbins, Cincinnati Children's Hospital). The 8.2-kb DNA fragment was microinjected as a transgene into pronuclei of eggs from BDF1 mice. The eggs were then transferred into the oviducts of pseudopregnant ICR mice. The transgenic founders were identified by Southern blot and PCR analysis. Line 2–5 and Line 9–12 were established and maintained by breeding to C57BL/6 mice. Line 9–12 was selected for further analysis on the basis of a higher level of transgene expression.

**BMMNC infusion and CM injection.** BMMNC (2.0×10^7^) isolated from a male wild-type mouse and suspended in 200 µl of PBS or an equal volume of PBS as a control were injected into the tail veins of anesthetized (4% inhaled isoflurane) 8-week-old male EGFRdn mice and 11-week-old male DOX and OMI mice. CM (200 µl) from Gr-1(+) cells from male wild-type mice or isovolume serum-depleted DMEM were infused into the tail veins of anesthetized 8-week-old male EGFRdn mice and 11-week-old male DOX mice under anesthesia. Anti-mouse insulin-like growth factor-1 (IGF-1) (0.1 µg/g body weight) or anti-goat immunoglobulin G (IgG) (0.1 µg/g body weight) antibodies were intraperitoneally injected into 11-week-old male DOX mice 2 h before CM infusion. Anti-activin A (20 µg) or anti-mouse IgG (20 µg) antibodies were intraperitoneally injected at 48-h intervals into male EGFRdn mice from 10 to 12 weeks of age. Pegvisomant (10 mg/kg body weight) or vehicle (control) were intraperitoneally injected into 8-week-old male DOX mice 30 min before CM infusion.

**Evaluation of cell shortening and the beating rate of cardiomyocytes.** After 12 h starvation with 500 µl serum-depleted DMEM in 12-well dishes, rat cardiomyocytes were cultured with 500 µl of CM or serum-depleted DMEM. At specific times, the cultured cardiomyocytes were video recorded for 10 sec, and the percentage of cell shortening was analyzed using ImageExpress version 5.5 (Nippon Roper). To measure the percentage of cell shortening, two regions of interest were fixed by the software, which analyzed the beating distance of a single cardiomyocyte, and divided the distance by the length between the regions of interest. The number of beats of single cardiomyocyte was counted for 10 sec to determine the beating rate. For antibody treatment *in vitro*, the starved cardiomyocytes were pretreated with anti-IGF-1 (10 µg/ml) or anti-goat IgG (10 µg/ml) antibodies for 2 h before adding CM. For pegvisomant treatment *in vitro*, the cardiomyocytes were pretreated with pegvisomant (12.5 µg/ml) for 30 min before adding CM.

**Echocardiography and catheterization.** Transthoracic echocardiographic analysis and catheterization analysis were performed as previously described [11]_ENREF_9. Briefly, the +dp/dt in the left ventricle was measured using a catheter, which was introduced retrogradely *via* the carotid artery.

**Cell isolation.** Neonatal rat cardiomyocytes were isolated and separately collected as described previously [43]. Cardiomyocytes were plated at a density of 1×10^5^ cells/cm^2^ on six-, 12- and 24-well dishes (BD Falcon) coated with 1% gelatin and cultured in DMEM supplemented with 10% FBS. Adult cardiomyocytes were prepared as previously described [44]. BMMNC and PBMNC were isolated from 8-week-old male C57BL/6, male GFP mice, and male EGFRdn mice by density gradient centrifugation with Histopaque-1083, as previously described [45]. PBMNC were also isolated from human subjects, as previously described [46].

**Sorting of harvested BMMNC into sub-populations and collection of CM.** After BMMNC were harvested from male wild-type mice, the cells were sorted into Gr-1(+) cells, B220(+) cells, TER(+) cells, and lineage-negative populations using a Magnetic Cell Sorting system (Miltenyi Biotec), as previously described [47]. To collect the CM, the individual sub-populations were seeded onto 24-well dishes with 200 µl of serum-depleted DMEM. After incubation for 24 h in serum-depleted DMEM, the supernatant (CM) was collected, and any cells were removed by filtering through a 0.45-µm filter (BD Falcon).

**Phase-contrast live imaging.** Live images of beating cardiomyocytes were taken using a Leica inverted microscope (Leica) equipped with a phase-contrast objective and a CCD camera (Leica).

**Flow cytometry.** The percentage of cells expressing each cell surface antigen was analyzed using a FACSCalibur (Becton Dickinson Immunocytometry Systems) and Cell Quest Pro version 5.2 software.

**RNA extraction and DNA microarray analysis.** Total RNA was extracted from 12-week-old male wild-type (*n* = 4) and age-matched male EGFRdn mice (*n* = 4) using a RNeasy Mini Kit (Qiagen) according to the manufacturer's protocol. RNA quality was assessed with an Agilent 2100 Bioanalyzer (Agilent Technologies). cRNA preparation, fragmentation, hybridization, and scanning of a GeneChip® Mouse Genome 430 2.0 Arrays (Affymetrix) were performed according to the manufacturer's protocol. cRNA was labeled using a Two-cycle Eukaryotic Target Labeling assay with a GeneChip Expression 3′ amplification two-cycle labeling and control reagents kit (Affymetrix). Briefly, cDNA was generated from total RNA (100 ng) using SuperScript II (Invitrogen) and a T7-oligo(dT) promoter primer (Affymetrix). After second-strand cDNA synthesis, cDNA was converted to cRNA by an *in vitro* transcription reaction (MEGAscript T7 kit, Ambion). The cRNA was then purified using a Sample Cleanup Module (Affymetrix), and the yield was monitored with a spectrophotometer. The second cycle of cDNA synthesis was performed, followed by the same cleanup as above and a second *in vitro* transcription reaction cycle with biotin-labeled ribonucleotides and T7 RNA polymerase. The labeled cRNA was purified, using a Sample Cleanup Module and denatured at 94°C before hybridization. The samples were hybridized to GeneChip® Mouse Genome 430 2.0 Arrays at 45°C for 16 h with rotation at 60 rpm. The arrays were then washed, stained with phycoerythrin–streptavidin (Molecular Probes), washed, and scanned with a GeneChip Scanner 3000 7G (Affymetrix). The data were analyzed with GeneSpring version 7.3.1 software (Agilent Technologies).

**Reverse transcriptase-PCR.** RNA extraction and RT-PCR were performed as previously described [11]. Real-time PCR amplification was performed using an Applied Biosystems 7500 real-time PCR system (Applied Biosystems) with QuantiTect SYBR Green PCR Master Mix (Qiagen). The PCR protocol comprised an initial denaturation step (94°C, 15 sec) followed by 60 cycles of amplification and quantification (55°C for 30 sec and 72°C for 35 sec) and a melting curve program (60–95°C). The relative mRNA expression level was calculated using the standard curve of GAPDH. All samples were independently analyzed at least three times for each gene. Semi-quantitative RT-PCR of GH was performed using 0.4 µg of total RNA and followed by 40 cycles of the above conditions. The primer sequences were QT00311654 (Qiagen) for GH in real-time PCR, 5′-TCCTGTGGACAGATCACTGC-3′ and 5′-AATGTAGGCACGCTCGAACT-3′ for GH in semi-quantitative PCR, QT00309099 (Qiagen) for GAPDH, and 5′-GGACCTGGCTGGCCGGGACC-3′ and 5′-GCGGTGCACGATGGAGGGGC-3′ for β-actin. For semi-quantitative RT-PCR, the PCR products were size-fractionated by 2% agarose gel electrophoresis.

**Northern blot analysis.** For northern blot analysis, total RNA (20 µg) was extracted from hearts using TRIzol Reagent (Invitrogen) and hybridized with a cDNA probe for *EGFRdn*. 18S rRNA ethidium bromide staining was used to quantify RNA loading.

**Analysis of phosphorylated ErbB receptor expression.** Four-week-old mice were anesthetized by intraperitoneal injection of urethane (2 mg/g body weight) followed by intravenous injection of HB-EGF (0.5 µg/g body weight, R&D Systems), NRG-1β (0.5 µg/g body weight, R&D Systems), or vehicle *via* the inferior vena cava. After 5 min, the hearts were immediately excised and homogenized in a buffer containing 50 mmol/l HEPES (pH 7.5), 137 mmol/l NaCl, 1 mmol/l MgCl_2_, 1 mmol/l CaCl_2_, 10 mmol/l Na-pyrophosphate, 2 mmol/l EDTA, 1% NP-40, 10% glycerol, 2 mmol/l Na_3_VO_4_, 10 mmol/l NaF, and protease inhibitor cocktail (Complete Mini, Roche Applied Science). To analyze the tyrosine phosphorylation of ErbB receptors, equivalent amounts of proteins were subjected to immunoprecipitation with the specific antibodies, fractionated by 6% SDS-PAGE, and immunoblotted with the mouse monoclonal anti-phosphotyrosine antibody 4G10 (Millipore). Horseradish peroxidase-conjugated anti-mouse IgG antibody (GE Healthcare) was used as the secondary antibody, and the bound antibodies were detected using an ECL detection kit (GE Healthcare).

**ELISA.** Serum and CM concentrations of cAMP, GH and activin A were measured by ELISA (cAMP and activin A, R&D Systems; GH, LINCO Research). To prepare cell lysates for cAMP analysis, cardiomyocytes were seeded (4×10^5^ cells/cm) onto six-well dishes coated with 1% gelatin and cultured in DMEM supplemented with 10% FBS. After 5 d, the cells were washed three times with PBS and the medium was changed to serum-depleted DMEM. After incubation for 12 h in the serum-depleted medium, the cells were washed three times with PBS and the medium was replaced with 1 ml of serum-depleted DMEM with CM (1 ml), 2 ml of serum-depleted DMEM with 500 pg/ml GH, 2 ml of serum-depleted DMEM with 12.5 µg/ml pegvisomant, or 1 ml of serum-depleted DMEM plus 1 ml of CM and 12.5 µg/ml pegvisomant. Thirty minutes later, the cardiomyocytes were resuspended in lysis buffer in six-well dish.

To examine the expression of NFκB and phosphorylated NFκB in PBMNC, PBMNC isolated from wild-type male mice were cultured with AngII or TNFα. Thirty minutes later, PBMNC were resuspended in lysis buffer and the expression of NFκB and phosphorylated NFκB were examined using sandwich ELISA kits (Cell Signaling). Some cells were also treated with 50 µM NFκB p65 (Ser276) inhibitory peptide to inhibit NFκB activity.

**Western blot analysis.** Whole-cell lysates (30–50 µg) were resolved by SDS-PAGE. The separated proteins were transferred to a PVDF membrane (GE Healthcare) and incubated with the primary antibody, followed by an anti-IgG-horseradish peroxidase-conjugated secondary antibody. Proteins were detected using an ECL-Plus kit (GE Healthcare).

**Immunohistology.** The hearts were fixed with 4% paraformaldehyde and embedded in paraffin, or fixed in 10% neutralized formalin and embedded in Tissue-Tek OCT cryo-embedding compound (Sakura Finetek). The specimens were sectioned (5 µm thick), and stained with hematoxylin/eosin or Masson trichrome.

**Evaluation of cardiac hypertrophy.** To evaluate the mean diameter of LV cardiomyocytes, the shortest diameter of each cardiomyocyte was measured in nucleated transverse sections stained with hematoxylin-eosin. Thirty cardiomyocytes in each LV were measured using an ocular micrometer disc with a linear scale at a magnification of 400×, and the average cardiomyocyte diameter was calculated for each specimen. Four hearts were measured in each group. The cell surface area of isolated neonatal and adult cardiomyocytes was measured by planimetry in 50 randomly selected cells per specimen.

**Immunofluorescence staining.** Immunostaining was performed as previously described [45]. Images were taken using a fluorescent microscopy (Leica) with LAS AF software (Leica).

**Human subjects.** We enrolled 10 subjects who were outpatients of Department of Cardiology of Tokyo Women's Medical University Hospital. We obtained 10 ml of whole blood from each patient. Half of the blood sample was used to measure the serum activin A concentration and the remaining blood was used to measure GH in CM after PBMNC isolation. All patients were receiving medical therapies and exhibited New York Heart Association class II symptoms. We also enrolled 11 healthy age- and body mass index-matched volunteers. Characteristics of the patients and healthy subjects are summarized in Table S1.

**Statistics.** Data are presented as means ± s.e.m. We examined differences between groups by Student's *t* test or analysis of variance followed by Bonferroni's correction to compare means. A value of *P*<0.05 was considered to be significant.

## Supporting Information

Figure S1 **Overexpression of EGFRdn inhibited the functional activation of endogenous ErbB receptors in a dominant-negative manner.** (A) Northern blot analysis for the transgene expression in hearts from wild-type and two different founder lines of EGFRdn mice (L2–5 and L9–12). (B) Tyrosine phosphorylation of ErbB receptors in hearts from wild-type and EGFRdn mice (L9–12) at 5 min after injection of HB-EGF. In wild-type mice, intravenous injection of HB-EGF enhanced cardiac tyrosine phosphorylation of EGFR, ErbB2 and ErbB4, which was abrogated in EGFRdn hearts. HB-EGF, heparin-binding EGF-like growth factor. (C) Tyrosine phosphorylation of ErbB receptors in hearts from wild-type and EGFRdn mice (L9–12) at 5 min after the injection of NRG-1β. NRG-1β induced tyrosine phosphorylation of ErbB2 and ErbB4 in wild-type hearts, but not in EGFRdn hearts. NRG-1, neuregulin-1.
(TIF)

Figure S2 **Echocardiographic analysis of DOX mice.** (A) Representative M-mode images of wild-type and DOX mice. (B) Left ventricular diastolic and systolic dimensions, and FS of 11-week-old DOX mice (*n* = 36) and age-matched wild-type mice (*n* = 10). LVDd, left ventricular diastolic dimension; LVDs, left ventricular systolic dimension. Data are means ± s.e.m.
(TIF)

Figure S3 **Analysis of cardiac hypertrophy.** (A) The shortest diameter of each cardiomyocyte (*n* = 30 per group). Lower photographs, H&E-stained tissue sections. Scale bar, 75 µm. (B) Surface area of isolated adult cardiomyocytes (*n* = 50 per group). Lower photographs, representative images. Scale bar, 75 µm. Data are means ± s.e.m.
(TIF)

Figure S4 **Flow cytometric analysis.** The left and right panels show the expression of each cell surface marker before and after magnetic sorting (MACS), respectively.
(TIF)

Figure S5 **Cardiac hypertrophy** ***in vitro*** **.** Upper graph, cell surface area of neonatal rat cardiomyocytes (*n* = 50); lower photographs, representative images of the cells. Cardiomyocytes were stained with sarcomeric α-actinin (red). Nuclei were stained with Hoechst 33258 (blue). Scale bars, 75 µm. Data are means ± s.e.m.
(TIF)

Figure S6 **Comparison of GH concentration.** GH concentration in CM from Gr-1(+) cells isolated from old myocardial infarction (OMI) mice and DOX mice (*n* = 5). Data are means ± s.e.m.
(TIF)

Figure S7 **BMMNC improve the cardiac function of OMI mice via the GH receptor.** (A) At 4 weeks after coronary ligation, BMMNC were infused via the tail vein. Pegvisomant (10 mg/kg body weight) or vehicle (control) was intraperitoneally injected into OMI mice 30 min before infusing BMMNC. BMMNC infusion improved FS and +dp/dt at 3 d after infusion and these improvements were inhibited by pegvisomant (n = 5). (B) Masson trichrome staining. Panels show representative images. Scale bars: 1 mm. Data are means ± s.e.m.
(TIF)

Figure S8 **Direct effects of GH in the CM from Gr-1(+) cells on cardiomyocytes.** CM from Gr-1(+) cells from wild-type mice was infused into DOX-treated wild-type mice (wild-DOX) or DOX-treated cardiac-specific STAT3dn mice (STAT3dn-DOX). Gr-1(+) cell-derived CM improved FS (left) and +dp/dt (right) in wild-DOX mice (*n* = 5) at 1 d after infusion, but not in STAT3dn-DOX (*n* = 5). Data are means ± s.e.m.
(TIF)

Figure S9 **Serum activin A concentrations** (*n* = 5). Data are means ± s.e.m.
(TIF)

Figure S10 **TNFα increases the secretion of activin A from PBMNC** ***via*** **NFκB.** (A) Activin A levels in CM from PBMNC were upregulated by treatment with TNFα (*n* = 5). (B) TNFα (50 ng/ml) activated NFκB in PBMNC (*n* = 5). Left, total NFκB; right, phosphorylated NFκB. (C) TNFα (50 ng/ml) -mediated upregulation of activin A in PBMNC was inhibited by treatment with the NFκB inhibitory peptide (*n* = 5). Isotype peptide was used as control. Data are means ± s.e.m.
(TIF)

Table S1 **Characteristics of human subjects.**
(PDF)
