# Supplementary figures and images for: A Crucial Role of Activin A-Mediated Growth Hormone Suppression in Mouse and Human Heart Failure
Source: PLoS One. 2011 Dec 28;6(12):e27901. doi: 10.1371/journal.pone.0027901 (PMC3247209; doi:10.1371/journal.pone.0027901)

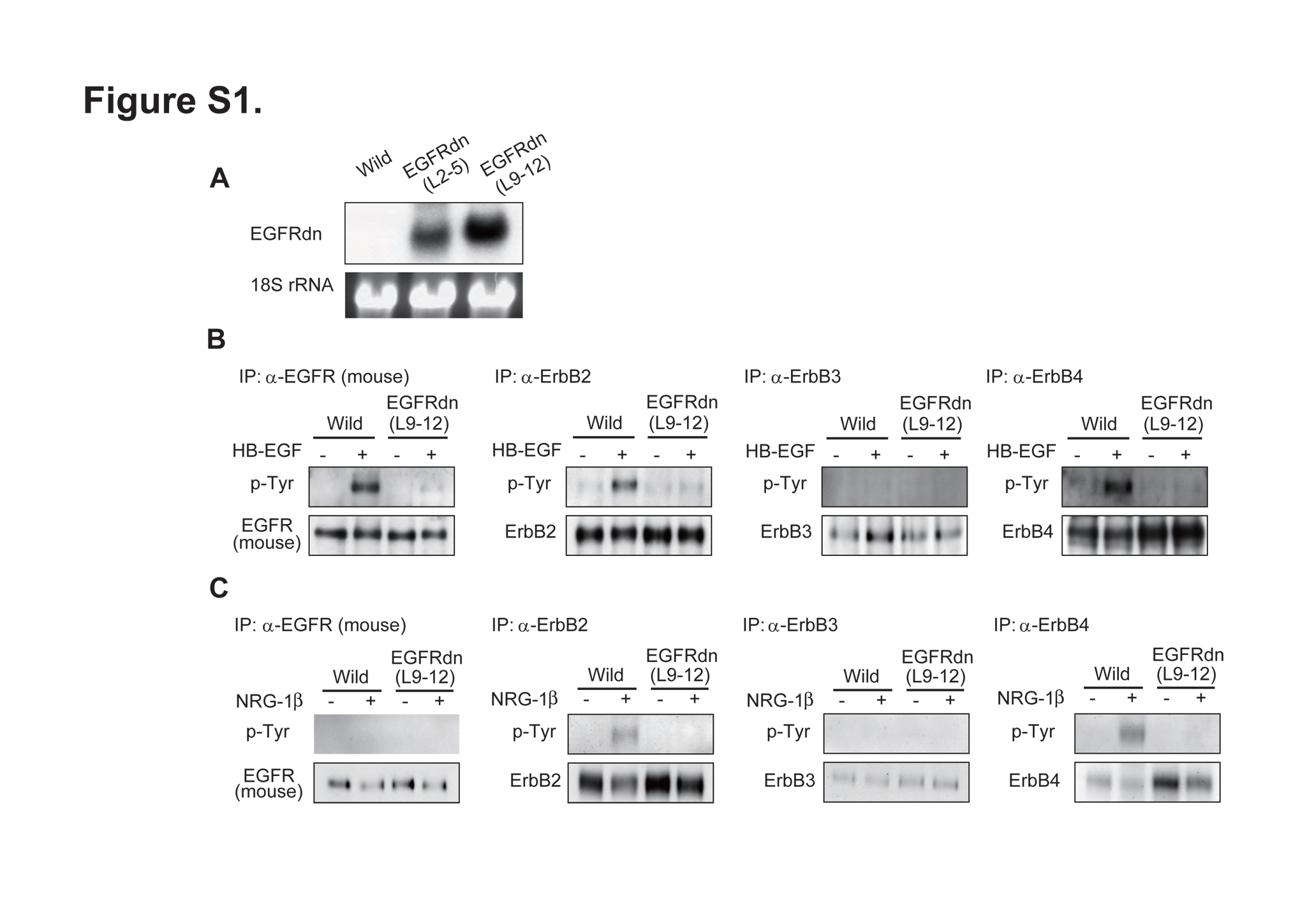

Supplement: Figure S1 — Overexpression of EGFRdn inhibited the functional activation of endogenous ErbB receptors in a dominant-negative manner. (A) Northern blot analysis for the transgene expression in hearts from wild-type and two different founder lines of EGFRdn mice (L2–5 and L9–12). (B) Tyrosine phosphorylation of ErbB receptors in hearts from wild-type and EGFRdn mice (L9–12) at 5 min after injection of HB-EGF. In wild-type mice, intravenous injection of HB-EGF enhanced cardiac tyrosine phosphorylation of EGFR, ErbB2 and ErbB4, which was abrogated in EGFRdn hearts. HB-EGF, heparin-binding EGF-like growth factor. (C) Tyrosine phosphorylation of ErbB receptors in hearts from wild-type and EGFRdn mice (L9–12) at 5 min after the injection of NRG-1β. NRG-1β induced tyrosine phosphorylation of ErbB2 and ErbB4 in wild-type hearts, but not in EGFRdn hearts. NRG-1, neuregulin-1. (TIF) [file pone.0027901.s001.tif]

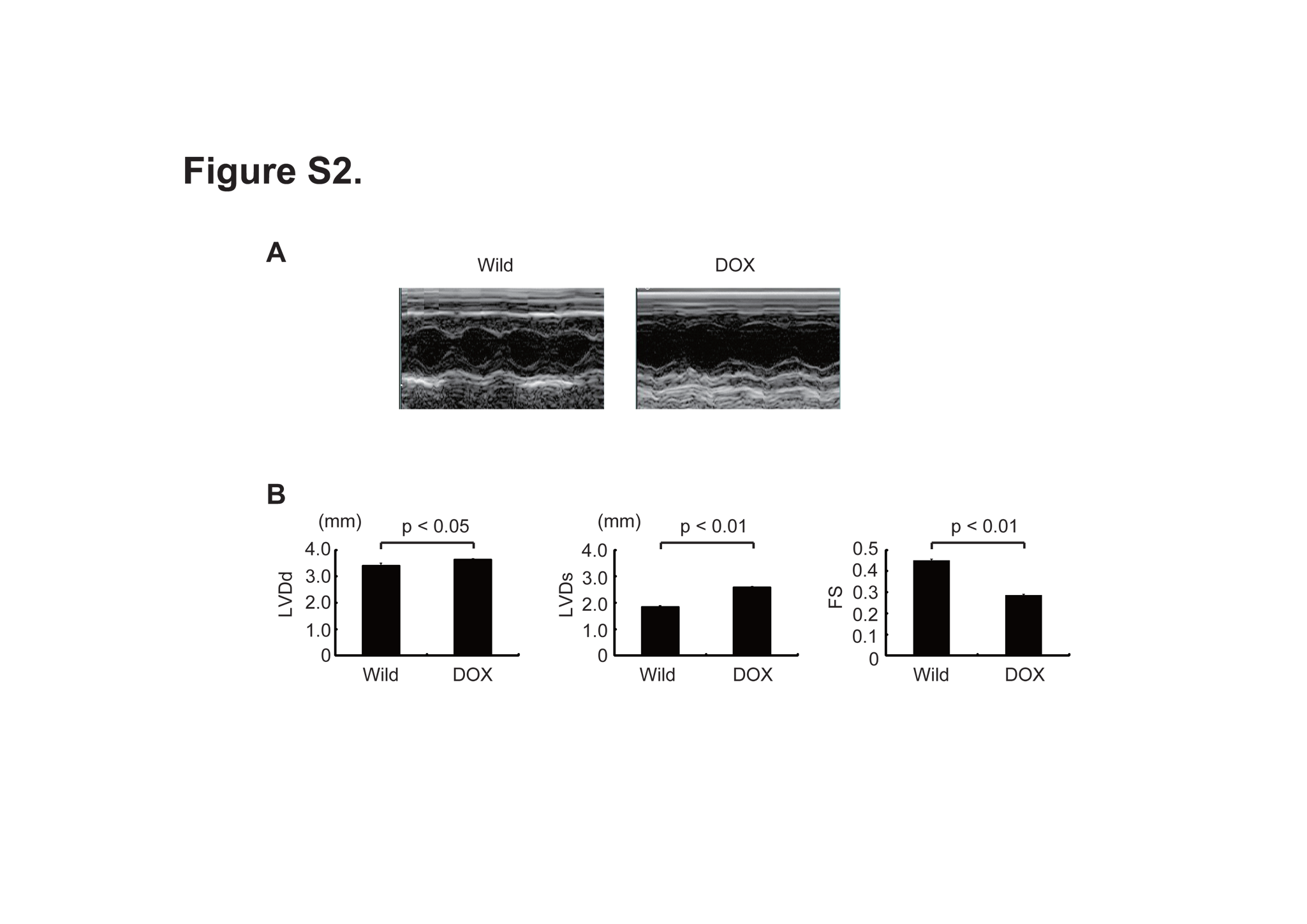

Supplement: Figure S2 — Echocardiographic analysis of DOX mice. (A) Representative M-mode images of wild-type and DOX mice. (B) Left ventricular diastolic and systolic dimensions, and FS of 11-week-old DOX mice (n = 36) and age-matched wild-type mice (n = 10). LVDd, left ventricular diastolic dimension; LVDs, left ventricular systolic dimension. Data are means ± s.e.m. (TIF) [file pone.0027901.s002.tif]

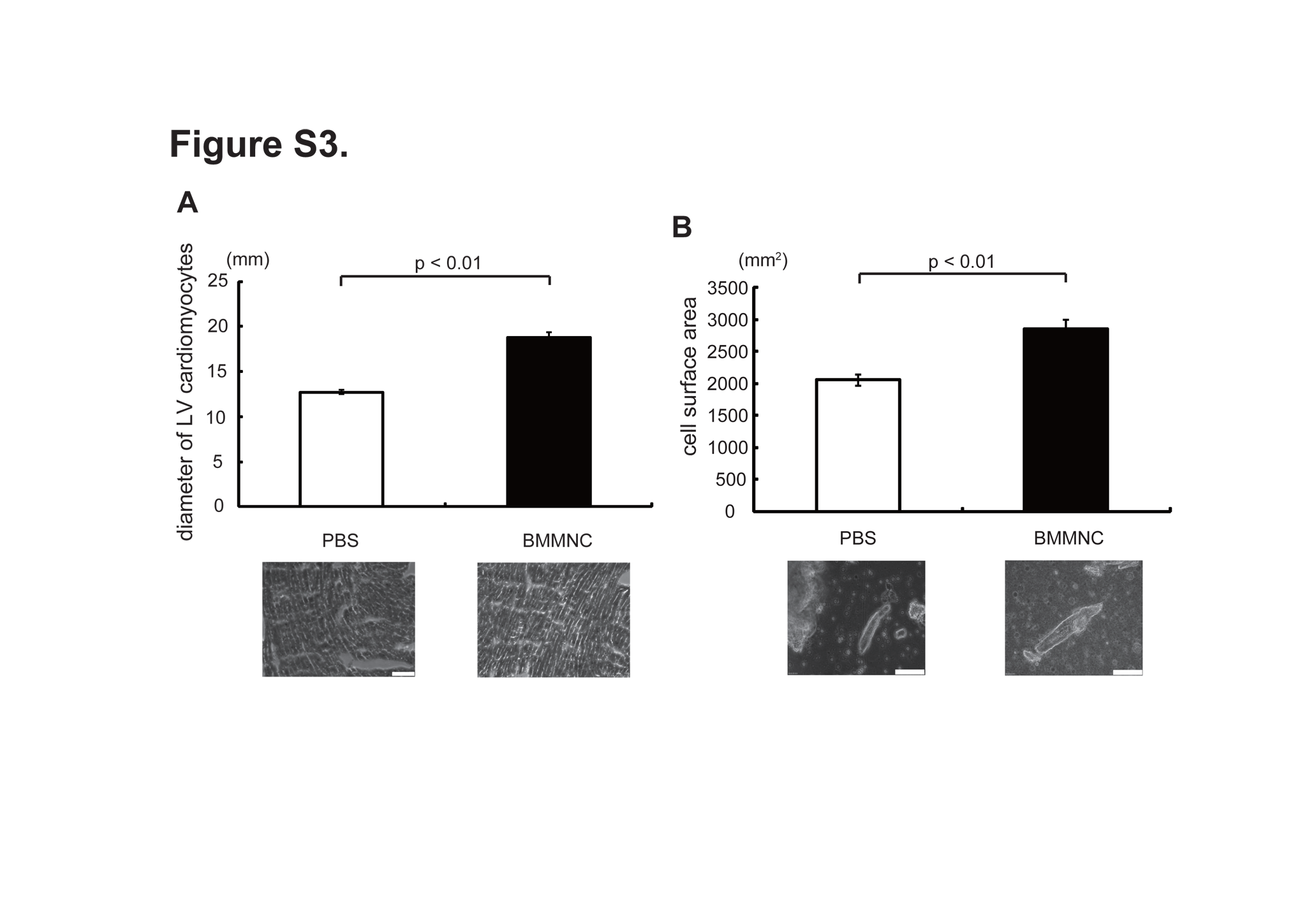

Supplement: Figure S3 — Analysis of cardiac hypertrophy. (A) The shortest diameter of each cardiomyocyte (n = 30 per group). Lower photographs, H&E-stained tissue sections. Scale bar, 75 µm. (B) Surface area of isolated adult cardiomyocytes (n = 50 per group). Lower photographs, representative images. Scale bar, 75 µm. Data are means ± s.e.m. (TIF) [file pone.0027901.s003.tif]

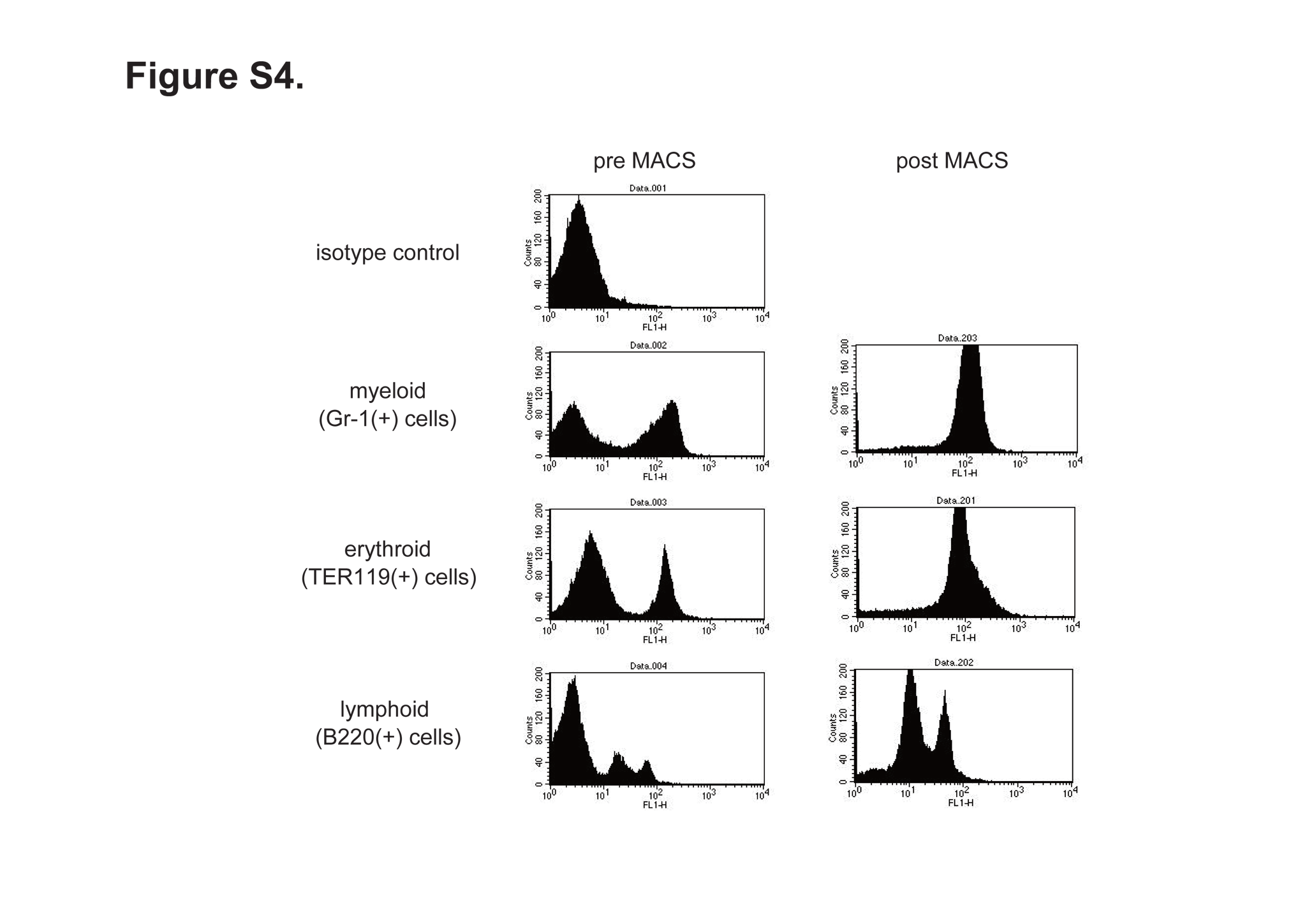

Supplement: Figure S4 — Flow cytometric analysis. The left and right panels show the expression of each cell surface marker before and after magnetic sorting (MACS), respectively. (TIF) [file pone.0027901.s004.tif]

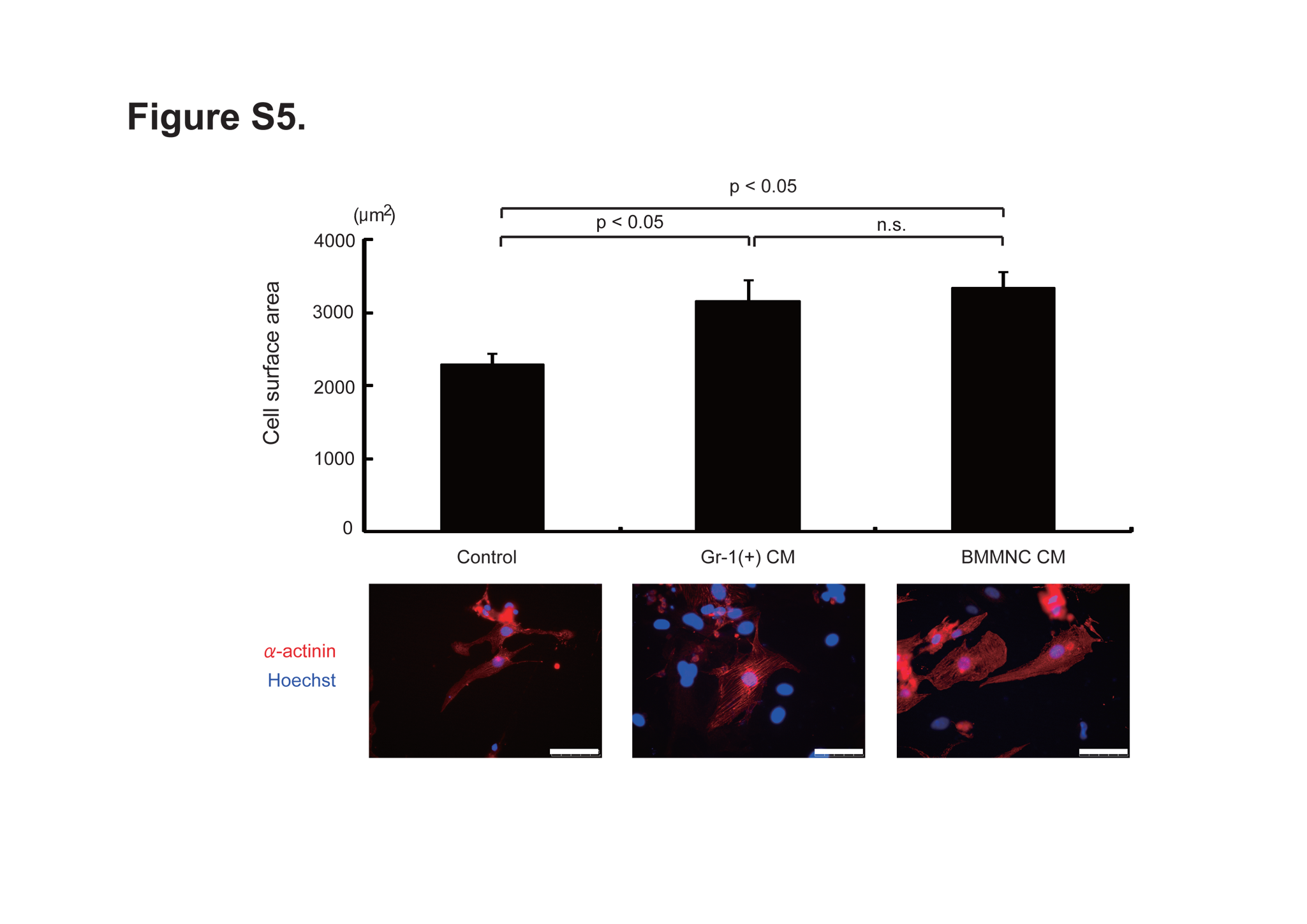

Supplement: Figure S5 — Cardiac hypertrophy in vitro . Upper graph, cell surface area of neonatal rat cardiomyocytes (n = 50); lower photographs, representative images of the cells. Cardiomyocytes were stained with sarcomeric α-actinin (red). Nuclei were stained with Hoechst 33258 (blue). Scale bars, 75 µm. Data are means ± s.e.m. (TIF) [file pone.0027901.s005.tif]

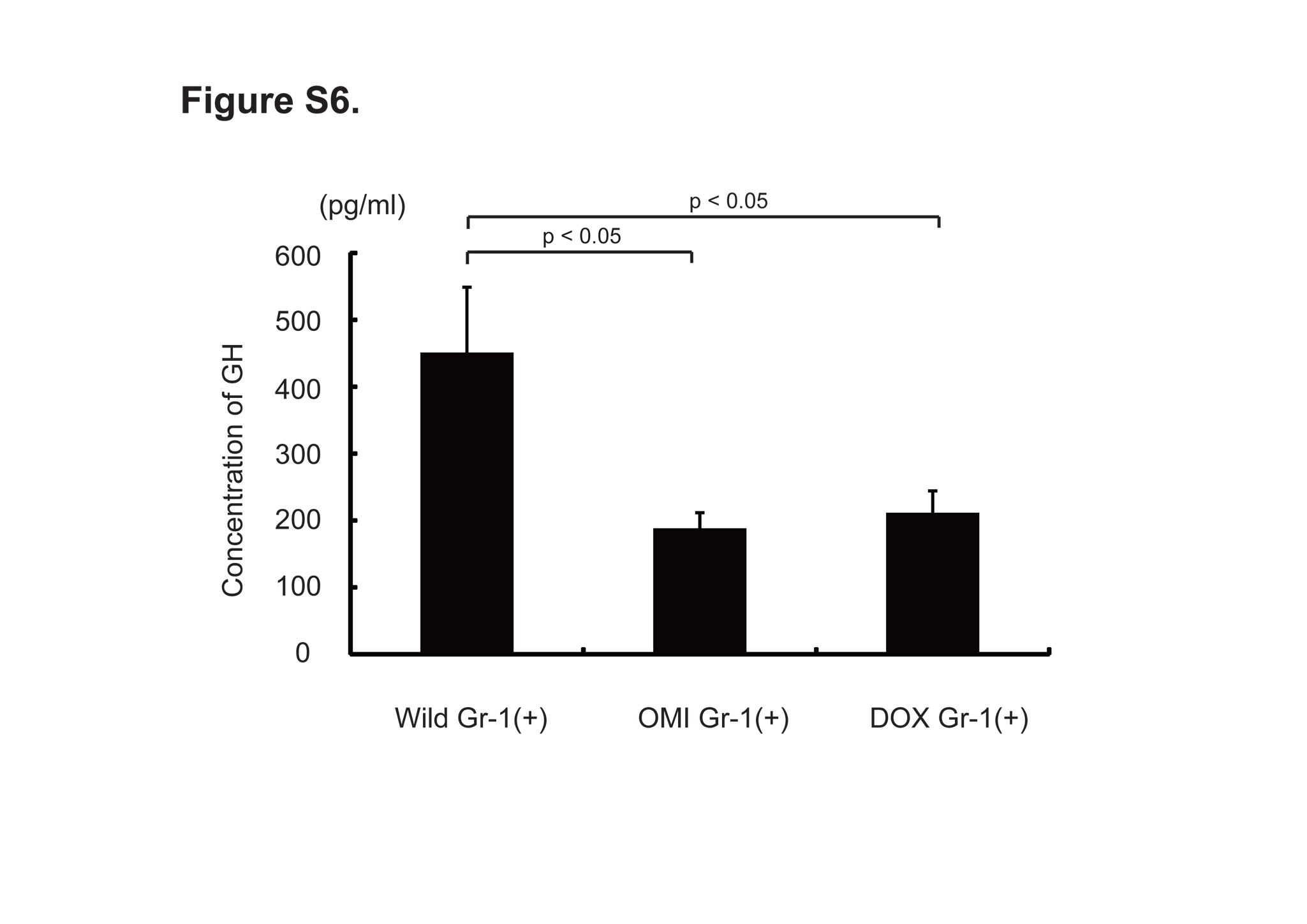

Supplement: Figure S6 — Comparison of GH concentration. GH concentration in CM from Gr-1(+) cells isolated from old myocardial infarction (OMI) mice and DOX mice (n = 5). Data are means ± s.e.m. (TIF) [file pone.0027901.s006.tif]

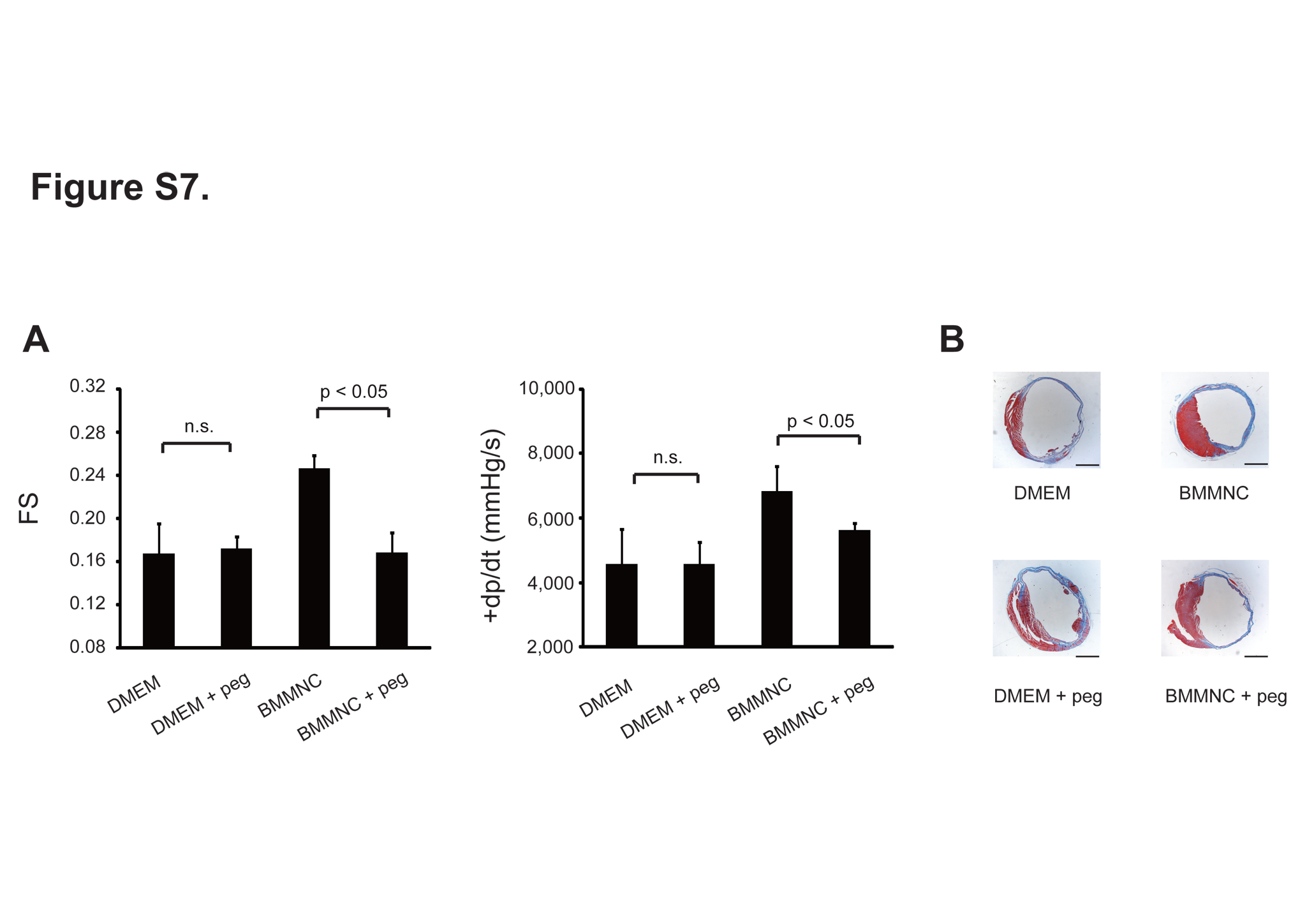

Supplement: Figure S7 — BMMNC improve the cardiac function of OMI mice via the GH receptor. (A) At 4 weeks after coronary ligation, BMMNC were infused via the tail vein. Pegvisomant (10 mg/kg body weight) or vehicle (control) was intraperitoneally injected into OMI mice 30 min before infusing BMMNC. BMMNC infusion improved FS and +dp/dt at 3 d after infusion and these improvements were inhibited by pegvisomant (n = 5). (B) Masson trichrome staining. Panels show representative images. Scale bars: 1 mm. Data are means ± s.e.m. (TIF) [file pone.0027901.s007.tif]

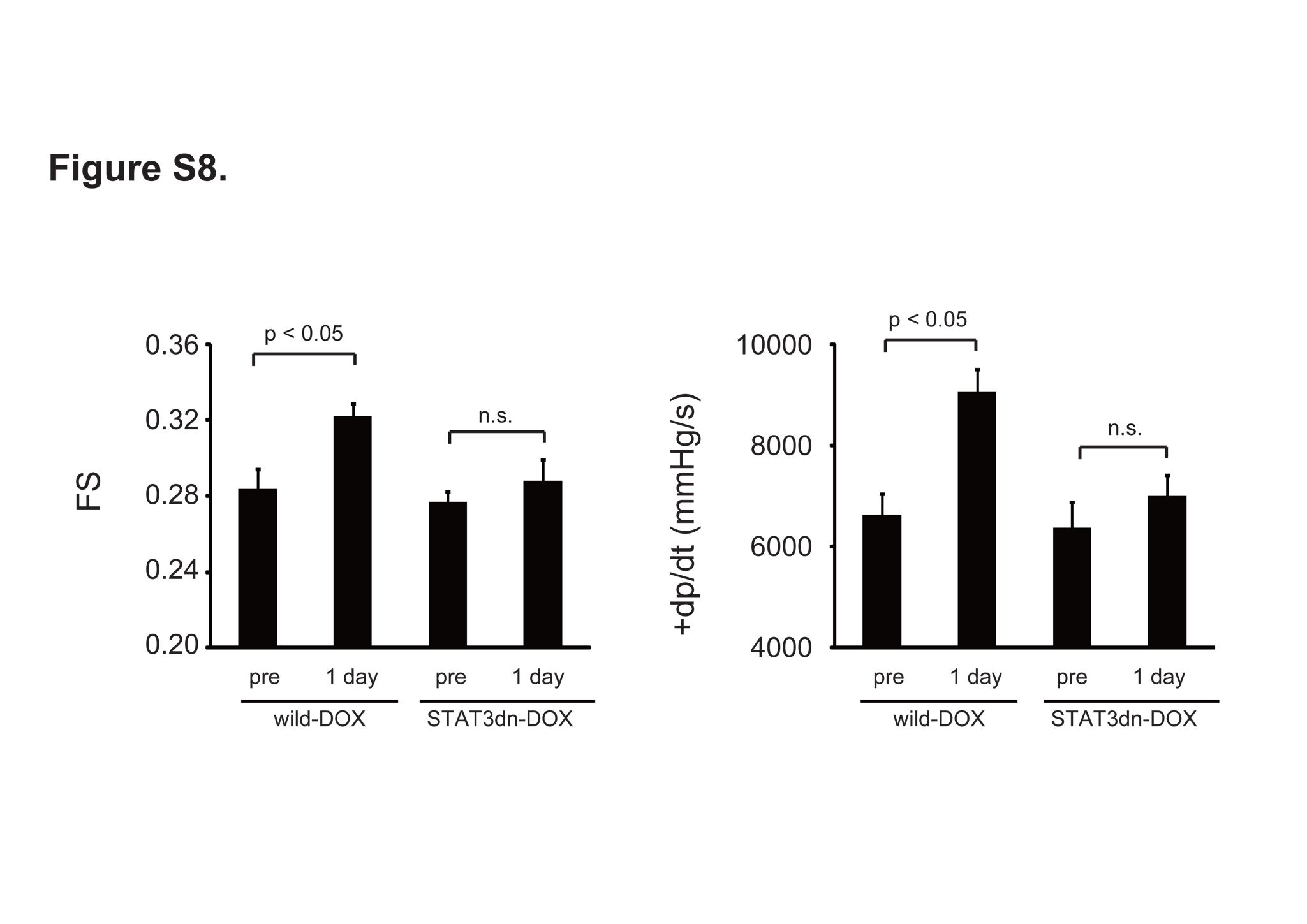

Supplement: Figure S8 — Direct effects of GH in the CM from Gr-1(+) cells on cardiomyocytes. CM from Gr-1(+) cells from wild-type mice was infused into DOX-treated wild-type mice (wild-DOX) or DOX-treated cardiac-specific STAT3dn mice (STAT3dn-DOX). Gr-1(+) cell-derived CM improved FS (left) and +dp/dt (right) in wild-DOX mice (n = 5) at 1 d after infusion, but not in STAT3dn-DOX (n = 5). Data are means ± s.e.m. (TIF) [file pone.0027901.s008.tif]

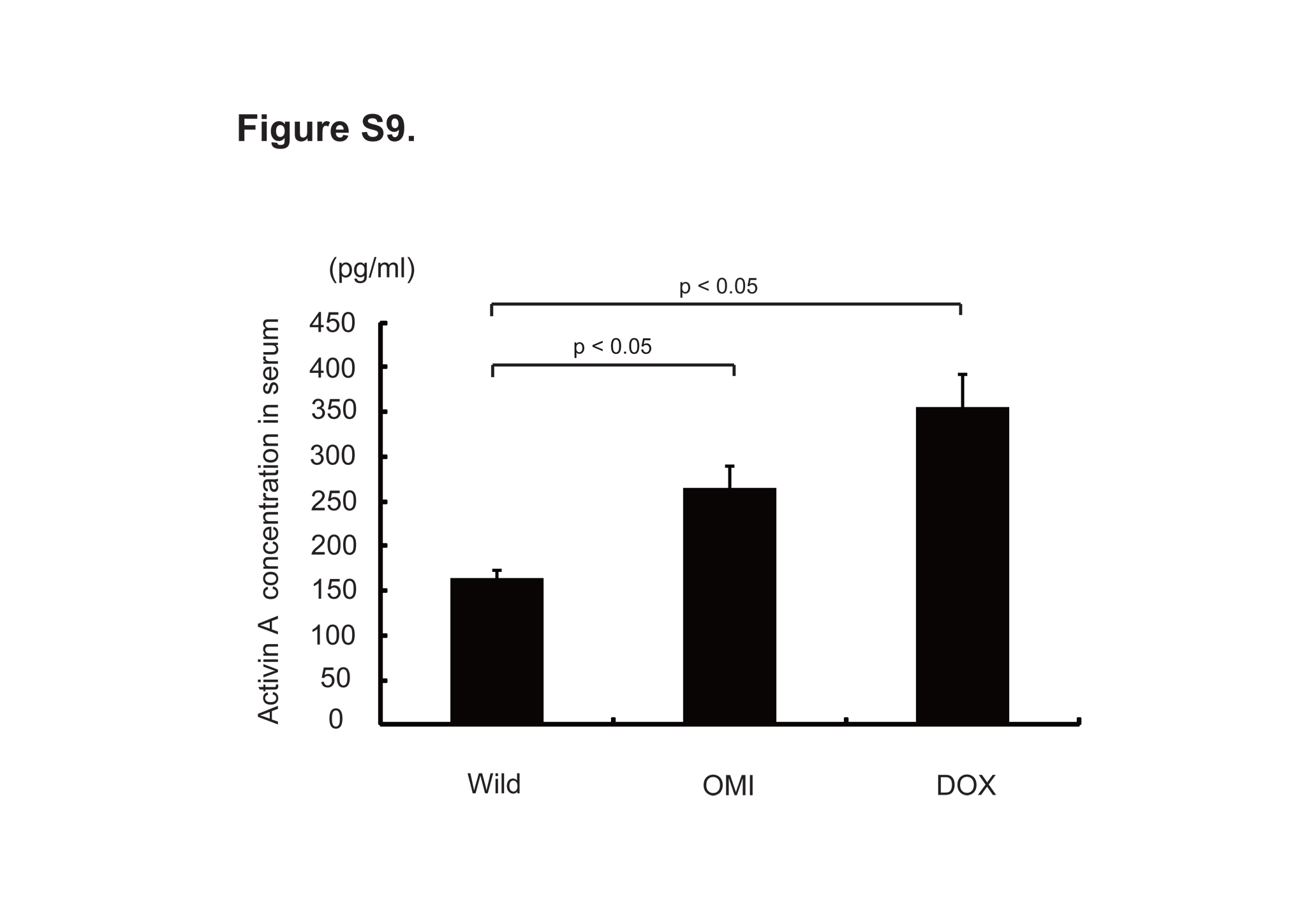

Supplement: Figure S9 — Serum activin A concentrations (n = 5). Data are means ± s.e.m. (TIF) [file pone.0027901.s009.tif]

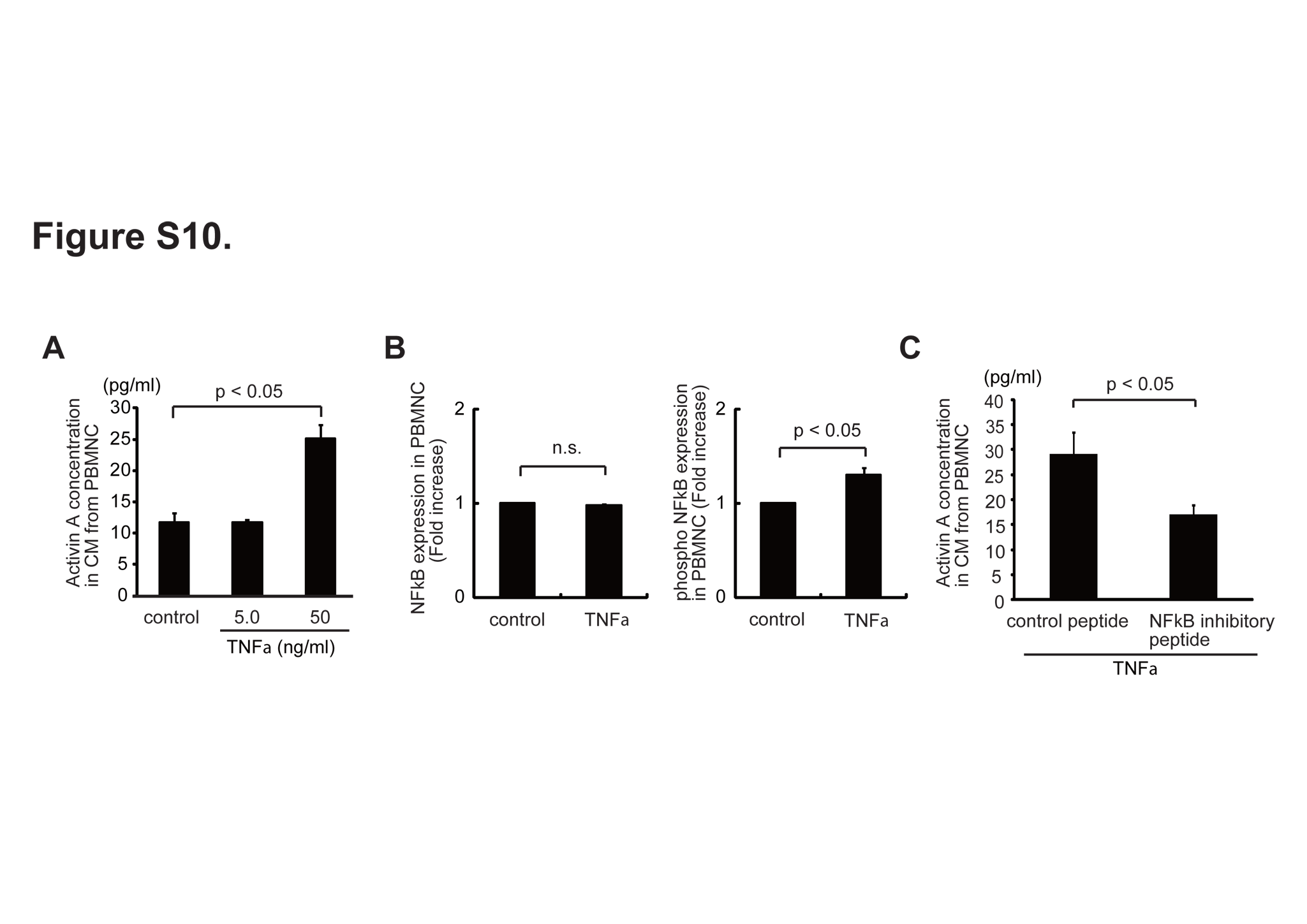

Supplement: Figure S10 — TNFα increases the secretion of activin A from PBMNC via NFκB. (A) Activin A levels in CM from PBMNC were upregulated by treatment with TNFα (n = 5). (B) TNFα (50 ng/ml) activated NFκB in PBMNC (n = 5). Left, total NFκB; right, phosphorylated NFκB. (C) TNFα (50 ng/ml) -mediated upregulation of activin A in PBMNC was inhibited by treatment with the NFκB inhibitory peptide (n = 5). Isotype peptide was used as control. Data are means ± s.e.m. (TIF) [file pone.0027901.s010.tif]
