# Supplementary material for: A Crucial Role of Activin A-Mediated Growth Hormone Suppression in Mouse and Human Heart Failure
Source: PLoS One. 2011 Dec 28;6(12):e27901. doi: 10.1371/journal.pone.0027901 (PMC3247209; doi:10.1371/journal.pone.0027901)
Supplement: Table S1 — Characteristics of human subjects. (PDF) [file pone.0027901.s011.pdf]

Table S1. Characteristics of human subjects.

	DCM patients n = 10	Healthy subjects n = 11
Age	53 ± 13	44 ± 10
Sex (M/F), n	9/1	10/1
BMI	25 ± 4	23 ± 3
LVEF	39 ± 12	n.d.
BNP	52 ± 49	n.d.
NYHA class (I/II/III/IV), n	0/10/0/0	n.d.
Medication (%)		
ACE inhibitor or ARB	100	0
β-blocker	100	0
Aldosteron receptor blocker	50	0
Diuretics	70	0
Digoxin	30	0

BMI, body mass index; LVEF, left ventricular ejection fraction; BNP, B-type natriuretic peptide; NYHA, New York Heart Association; ACE, angiotensin II converting enzyme; ARB, angiotensin II receptor blocker; n.d., not done.
